# Supplementary material for: Ultrasound-activated prodrug-loaded liposome for efficient cancer targeting therapy without chemotherapy-induced side effects
Source: J Nanobiotechnology. 2024 Jan 3;22:2. doi: 10.1186/s12951-023-02195-5 (PMC10763105; doi:10.1186/s12951-023-02195-5)
Supplement: Supplementary file 1 — Supplementary Material 1: Experimental section [file 12951_2023_2195_MOESM1_ESM.docx]

**Ultrasound-Activated Prodrug-Loaded Liposome for Efficient Cancer Targeting Therapy without Chemotherapy-Induced Side Effects**

Yifan Jiang,^1, 2, †^ Hongjian Chen,^1, 3^^,^ ^†^ Tao Lin,^1, †^ Chao Zhang,^1, 2^ Jiaxin Shen,^1, 2^ Jifan Chen,^1, 2^ Yanan Zhao,^1^ Wen Xu,^1^ Guowei Wang, ^1, 2, *^ and Pintong Huang ^1, 2, 3, *^

^1^ Department of Ultrasound in Medicine, The Second Affiliated Hospital of Zhejiang University School of Medicine, Zhejiang University, Hangzhou 310009, China.

^2^ Research Center of Ultrasound in Medicine and Biomedical Engineering, The Second Affiliated Hospital of Zhejiang University School of Medicine, Zhejiang University, Hangzhou 310009, China.

^3^ Research Center for Life Science and Human Health, Binjiang Institute of Zhejiang University, Hangzhou 310053, China.

^†^ These authors contributed equally to this work.

^*^Corresponding author, E-mail: wangguowei@zju.edu.cn, and [huangpintong@zju.edu.cn](mailto:huangpintong@zju.edu.cn)

**ABSTRACT**

**Background**

Off-targeted distribution of chemotherapeutic drugs causes severe side effects, further leading to poor prognosis and patient compliance. Ligand/receptor-mediated targeted drug delivery can improve drug accumulation in the tumor but it always attenuated by protein corona barriers.

**Results**

To address these problems, a radically different strategy is proposed that can leave the off-targeted drugs inactive but activate the tumor-distributed drugs for cancer-targeting therapy in a tumor microenvironment-independent manner. The feasibility and effectiveness of this strategy is demonstrated by developing an ultrasound (US)-activated prodrug-loaded liposome (CPBSN38L) comprising the sonosensitizer chlorin e6 (Ce6)-modified lipids and the prodrug of pinacol boronic ester-conjugated SN38 (PBSN38). Once CPBSN38L is accumulated in the tumor and internalized into the cancer cells, under US irradiation, the sonosensitizer Ce6 rapidly induces extensive production of intracellular reactive oxygen species (ROS), thereby initiating a cascade amplified ROS-responsive activation of PBSN38 to release the active SN38 for inducing cell apoptosis. If some of the injected CPBSN38L is distributed into normal tissues, the inactive PBSN38 exerts no pharmacological activity on normal cells. CPBSN38L exhibited strong anticancer activity in multiple murine tumor models of colon adenocarcinoma and hepatocellular carcinoma with no chemotherapy-induced side effects, compared with the standard first-line anticancer drugs irinotecan and topotecan.

**Conclusions**

This study established a side-effect-evitable, universal, and feasible strategy for cancer-targeting therapy.

**Graphic abstract**


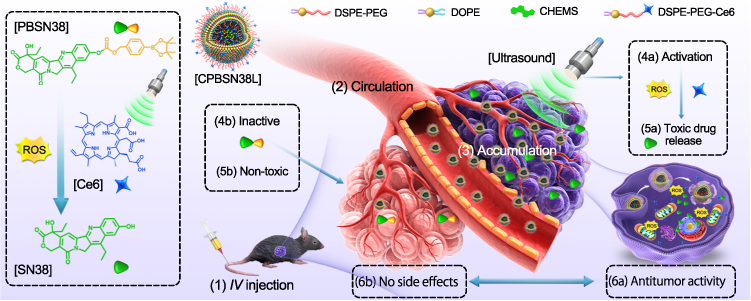


**KEYWORDS**: ultrasound, liposome, prodrug, stimuli-responsive drug delivery, cancer targeting therapy

**Introduction**

Solid tumors, such as hepatocellular carcinoma, colorectal cancer, pancreatic cancer, lung cancer, and breast cancer, are responsible for over 70% of the deaths in cancer patients.^[^[^1^](#_ENREF_1)^]^ New therapeutic approaches (e.g., immunotherapy and gene therapy) are remarkable clinical advancements in the treatment of various tumors.^[^[^2-5^](#_ENREF_2)^]^ A programmed cell death 1 inhibitor (e.g., pembrolizumab) demonstrates an eye-catching performance in melanoma and lung cancer treatments.^[^[^6^](#_ENREF_6)^,^ [^7^](#_ENREF_7)^]^ Engineered KRAS G12D mutant T cells exhibited impressive therapeutic potential against pancreatic cancer.^[^[^5^](#_ENREF_5)^]^ However, the clinical treatment of most solid tumors continues to mainly involve surgical resection combined with chemotherapy, because of the cost-effectiveness, safety, and efficacy of these methods, and the heterogeneity and penetration barriers of tumors.^[^[^8^](#_ENREF_8)^,^ [^9^](#_ENREF_9)^]^ Off-targeted chemotherapeutic drugs are often associated with serious side effects (e.g., emesis, alopecia, anemia, allergy, and bone marrow suppression), chemotherapy-related complications (e.g., liver and kidney damage, inflammation of the digestive system, and neurological disorders), and even death, which significantly reduce patient compliance and lead to poor prognosis.^[^[^10^](#_ENREF_10)^]^ Therefore, developing cancer treatments involving reduced side effects of chemotherapeutic drugs and having the ability to improve the quality of patient lives after chemotherapy in a simple, economic, universal, and efficient manner is of considerable significance.

The existing strategies of drug-loaded nanomedicines involve a preferential accumulation of active ingredients in the tumor while avoiding induction of off-target toxicity of normal tissues and organs by modifying nanocarriers with ligands.^[^[^11^](#_ENREF_11)^,^ [^12^](#_ENREF_12)^]^ These ligands can specifically identify and bind to the receptors typically overexpressed on cancer cells or tumor neovascular endothelial cells in the tumor microenvironment to achieve active target accumulation. Such active targeted drug delivery approaches have a notable impact in reducing the unwanted systemic toxicity of chemotherapy agents and improving antitumor efficacy, compared with the traditional strategy relying exclusively on passive targeting, which is well recognized as the enhanced permeability and retention (EPR) effect or transcytosis.^[^[^13-15^](#_ENREF_13)^]^ However, clinical outcomes of actively targeted therapies remain unsatisfactory against solid tumors, and the clinical translation of nanomedicine is progressing slowly.^[^[^16^](#_ENREF_16)^,^ [^17^](#_ENREF_17)^]^ A major cause is the adsorption of plasma proteins onto the nanoparticle surface, the so-called protein corona, which covers the targeting ligands and results in the loss of recognized specificity.^[^[^18^](#_ENREF_18)^,^ [^19^](#_ENREF_19)^]^ To overcome this dilemma, we previously developed a transcytosis-targeting peptide-decorated reconfigurable liposome. Ultrasonic cavitation can unravel surface plasma coronas on liposomal nanoparticles through ultrasound (US)-induced liposomal reassembly, thereby markedly restoring tumor targeting and achieving highly efficient tumor inhibition in multiple tumor models of patient-derived tumor xenograft.^[^[^20^](#_ENREF_20)^]^ Some latest studies have found that cancer treatments are associated with heterogeneous manifestation and tremendous variations in clinical therapeutic responses.^[^[^21^](#_ENREF_21)^,^ [^22^](#_ENREF_22)^]^ These studies have delineated that the abundance of specific subtypes and receptors varies widely in different cancer types, different patients with the same tumor type, and even within an individual tumor. This may reduce the ubiquity of clinical applications of ligand/receptor-mediated targeted nanomedicine. Thus, designing a cancer-targeting strategy in a tumor microenvironment-independent manner is acquisitive, so that it can be applied to a broad range of solid tumors.

US is an excellent external physical stimulation and produces biological effects in a high penetration depth, non-intrusive, and homogeneous manner. It is widely used in sonodynamic therapy, photoacoustic tomography, neuromodulation, and US-guided microbubble destruction for delivering cancer drugs.^[^^[23-27](#_ENREF_23)]^ According to some studies, the US can selectively cleave the disulfide or mechanochemical bonds for activating many small molecules in disease diagnosis and treatment.^[^[^28^](#_ENREF_28)^,^ [^29^](#_ENREF_29)^]^ We also previously reported a US-enhanced reactive oxygen species (ROS)-responsive charge-reversal polymeric nanocarrier for rapid delivery of cancer genes,^[^[^26^](#_ENREF_26)^]^ wherein low-intensity US could generate an excess of ROS to disequilibrate the chemical equilibrium of ROS and glutathione. We hypothesized that US-induced biological changes may be used as a universal trigger for developing targeted drug delivery systems that function in a tumor microenvironment-independent manner against various solid tumors without producing any side effects.

To overcome these aforementioned challenges, we here propose a different strategy wherein the off-targeted drugs remain inactive and the tumor-distributed drugs are activated for cancer-targeting therapy. The inactive off-targeted drugs remain inert and exert no side effects, whereas the universally activated drugs in the tumor eliminate the cancer cells. To demonstrate this strategy, we designed a US-activated prodrug-loaded liposome (CPBSN38L) composed of the sonosensitizer chlorin e6 (Ce6)-modified lipids and the prodrug pinacol boronic ester-conjugated 7-ethyl-10-hydroxycamptothecin (PBSN38) (Scheme 1A). CPBSN38L induces a US-initiated cascade amplified ROS-responsive activation of the PBSN38 prodrug-loaded liposomes with the aid of Ce6 (Scheme 1B). After being administered through intravenous injection, CPBSN38L stably circulates in the bloodstream and is inevitably distributed in the normal tissue. The non-toxic and unresponsive PBSN38 could reduce damage caused to normal tissues and avoid undesired side effects. A portion of CPBSN38L accumulates in the tumor through both the EPR effect and transcytosis and is subsequently internalized into the cancer cells. After US irradiation, the sonosensitizer Ce6 produces extensive amounts of intracellular ROS to trigger PBSN38 activation and release the active SN38, thereby inducing tumor cell apoptosis in a temporal- and spatial-controllable manner and realizing potent antitumor activity (Scheme 1C).


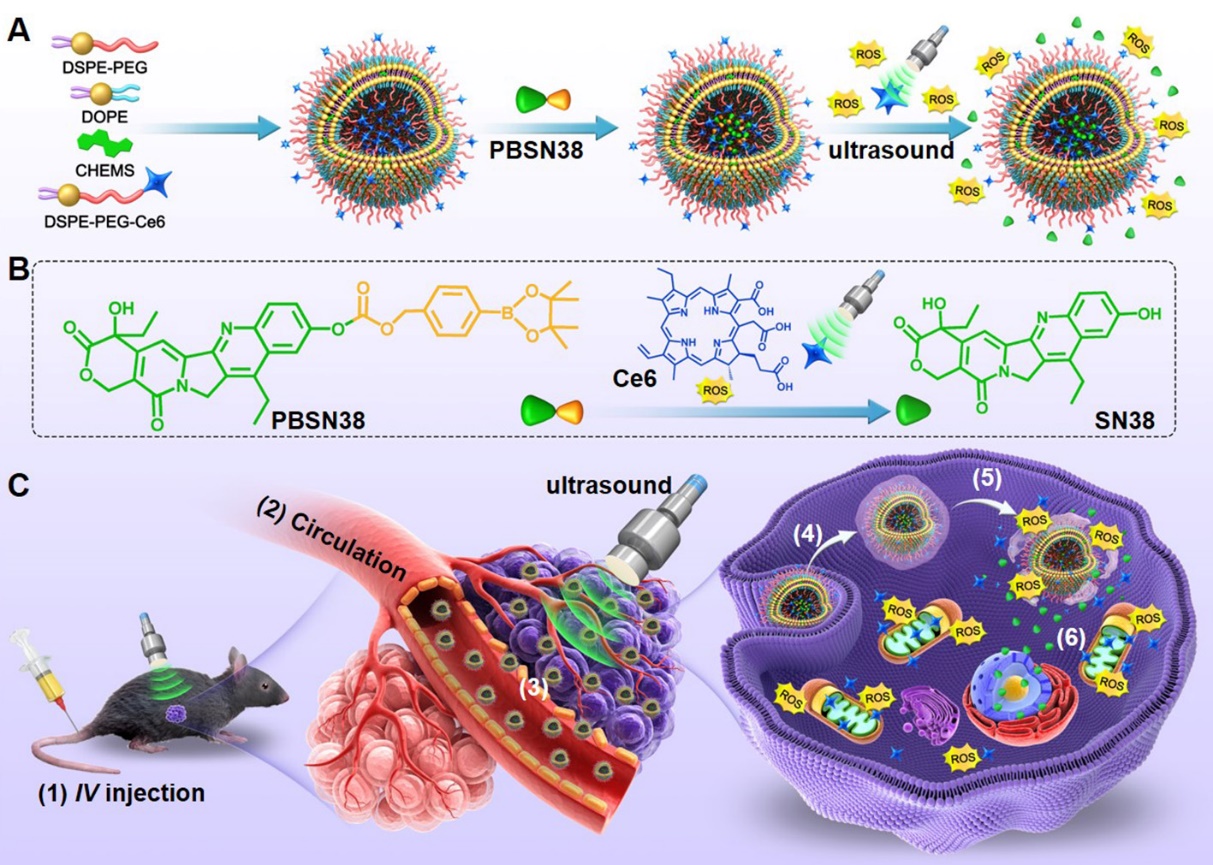


**Scheme 1**. Schematics of the US-activated prodrug-loaded liposome (CPBSN38L) and its delivery process for cancer-targeting therapy. **A** The composition, preparation, and transformability of CPBSN38L. **B** Chemical structure of the prodrug PBSN38 and Ce6- and US irradiation-induced activation of this prodrug. **C** After being administered through intravenous injection *(1)*, CPBSN38L circulates stably in the bloodstream *(2)*, accumulates in the tumor because of both the enhanced permeability and retention effect and transcytosis *(3),* and is internalized into the tumor cells *(4)*. After US irradiation, the sonosensitizer Ce6 produces large amounts of intracellular reactive oxygen species to trigger PBSN38 activation and release of the active SN38 *(5)*. The free SN38 induces cell apoptosis, thereby triggering strong anticancer activity in the murine tumor models of colon adenocarcinoma or hepatocellular carcinoma *(6)*.

**Results and Discussion**

**Synthesis and Characterization of Prodrugs**

7-Ethyl-10-hydroxycamptothecin (SN38), an active metabolite of irinotecan (CPT11), is 1,000-fold more cytotoxic in vitro than CPT11 and induces DNA strand break in cancer cells.^[^[^30^](#_ENREF_30)^]^ However, the clinical usage of SN38 is considerably limited because of its few disadvantages, such as low water solubility, poor stability, and potentially toxic side effects.^[^[^31^](#_ENREF_31)^]^ In this study, a new prodrug, PBSN38, was designed by conjugating SN38 to pinacol boronic ester through a carbonic ester linker. ROS can activate this prodrug to rapidly release the cytotoxic ingredient. Scheme S1A (Supporting Information) presents the process for synthesizing the ROS-responsive prodrug PBSN38. A ROS-insensitive prodrug phenylcarbinol ester-containing SN38 (PSN38) was also synthesized as a parallel control (Scheme S1B, Supporting Information). Both SN38 prodrugs were well-characterized using proton nuclear magnetic resonance, matrix-assisted laser desorption/ionization-time of flight mass spectrometry, and high-performance liquid chromatography (HPLC) (Fig. S1−S5, Supporting Information). To confirm that the designed prodrugs are ROS sensitive, PBSN38 and PSN38 were separately incubated with 5 mM H_2_O_2_ solution, with the prodrug and H_2_O_2_ at a 1% molar ratio. Under HPLC real-time monitoring, more than 90% of SN38 was released from PBSN38 within 1 h, whereas PSN38 released less than 2% of SN38 in 2 h (Fig. 1A and S5, Supporting Information), proving the ROS-responsiveness of PBSN38.

A suitable prodrug-response efficiency is a crucial factor for successfully constructing prodrug-loaded nanocarriers. This efficiency determines the sensitivity of cascade amplification drug release. A very high response efficiency may cause early and undesired drug activation, whereas a low response efficiency may induce no pharmacological actions. The PBSN38 activation capacity was further monitored through treatment with different H_2_O_2_ concentrations to determine the SN38 transformation rate (Fig. 1A). According to the HPLC results, the prodrug PBSN38 was transformed into SN38 after 2 h incubation at 5 mM H_2_O_2_. The prodrug transformation was less than 40% in a 0.5 mM H_2_O_2_ solution within 4 h. Negligible activation was observed in a 0.05 mM H_2_O_2_ solution. By contrast, less than 5% of PSN38 was activated with 5 mM H_2_O_2_ in 4 h, and almost no SN38 was produced at a lower H_2_O_2_ concentration. In normal cells, the H_2_O_2_ concentration is usually within two orders of magnitude from 1.0 nM up to a maximum of 0.7 mM.^[^[^32^](#_ENREF_32)^]^ At these concentration ranges for PBSN38 activation, little or no SN38 is released in normal cells. In general, cancer cells contain higher ROS levels than normal cells because the metabolic activity level is higher during tumorigenesis.^[^[^33^](#_ENREF_33)^]^ However, tumor cells utilize diverse adaptive strategies for maintaining a dynamic equilibrium that simultaneously allows tumor proliferation and avoids auto-oxidative stress, such as the oxidation-reduction equilibrium.^[^[^34^](#_ENREF_34)^]^ Thus, a ROS producer, the sonosensitizer Ce6 was selected and used in the following liposome fabrication.^[^[^23^](#_ENREF_23)^,^ [^24^](#_ENREF_24)^]^ US irradiation stimulated this sonosensitizer in tumor cells to generate large amounts of ROS for prodrug activation.

**Preparation and Characterization of Liposomes**

To fabricate the US-activated prodrug-loaded liposome (CPBSN38L), a Ce6-modified 1,2-distearyl-sn-glycerol-3-phosphoethanolamine-polyethylene glycol-2000 (DSPE-PEG-Ce6) lipid was selected and added to the liposomal formulas. To prepare CPBSN38L, the lipids of DSPE-PEG-Ce6, DOPE, DSPE-PEG, and CHEMS were mixed at a 1.5:1.5:1:1 mass ratio, followed by PBSN38 encapsulation with approximately 7% drug-loading capacity. TEM revealed that the resulting CPBSN38L was spherical, well-defined, monodispersed, and homogeneous with no appreciable aggregations. It had a particle size diameter of 36.3 ± 13.9 nm, a polydispersity index (PDI) of 0.2, and a zeta potential of −1.3 ± 0.2 mV (Fig. 1B). Two types of liposomes, namely the PBSN38-loaded liposome without the DSPE-PEG-Ce6 lipid (PBSN38L), and the Ce6-modified liposome without PBSN38 loading (CL) were correspondingly prepared as controls. They exhibited sizes and zeta potentials similar to those of CPBSN38L.

To assay the prodrug release ability of CPBSN38L, a PBS solution containing 10% FBS and 2% glycerol was used as a dialysate in the presence or absence of the H_2_O_2_ redox condition. Samples outside the dialysis bags were collected, and the drug concentration in the interval time was monitored through HPLC (Fig. 1C). Less than 20% of SN38 was released from the liposomes after 48h incubation in the PBS solution without H_2_O_2_, whereas SN38 was almost completely released at the same time with 5 mM H_2_O_2_. In addition to reducing the π-π stacking among PBSN38 via catalyzing phenylboronic acid pinacol ester, the presence of ROS can destabilize the liposome via oxidization of the lipids,^[^[^35^](#_ENREF_35)^,^ [^36^](#_ENREF_36)^]^ which collectively resulted in a higher SN38-releaseing in the presence of H_2_O_2_. This proved the good release ability of CPBSN38L under the H_2_O_2_ condition. A successive release of SN38 achieved the specific cascade amplification of cytotoxicity in cancer cells. Ce6 was activated by US irradiation to generate ROS, subsequently triggering SN38 release once the ester bond is cleaved. Treatment under US irradiation ensures sufficient cytotoxicity of SN38 in tumors but not in normal tissues, thereby possibly avoiding undesirable side effects.


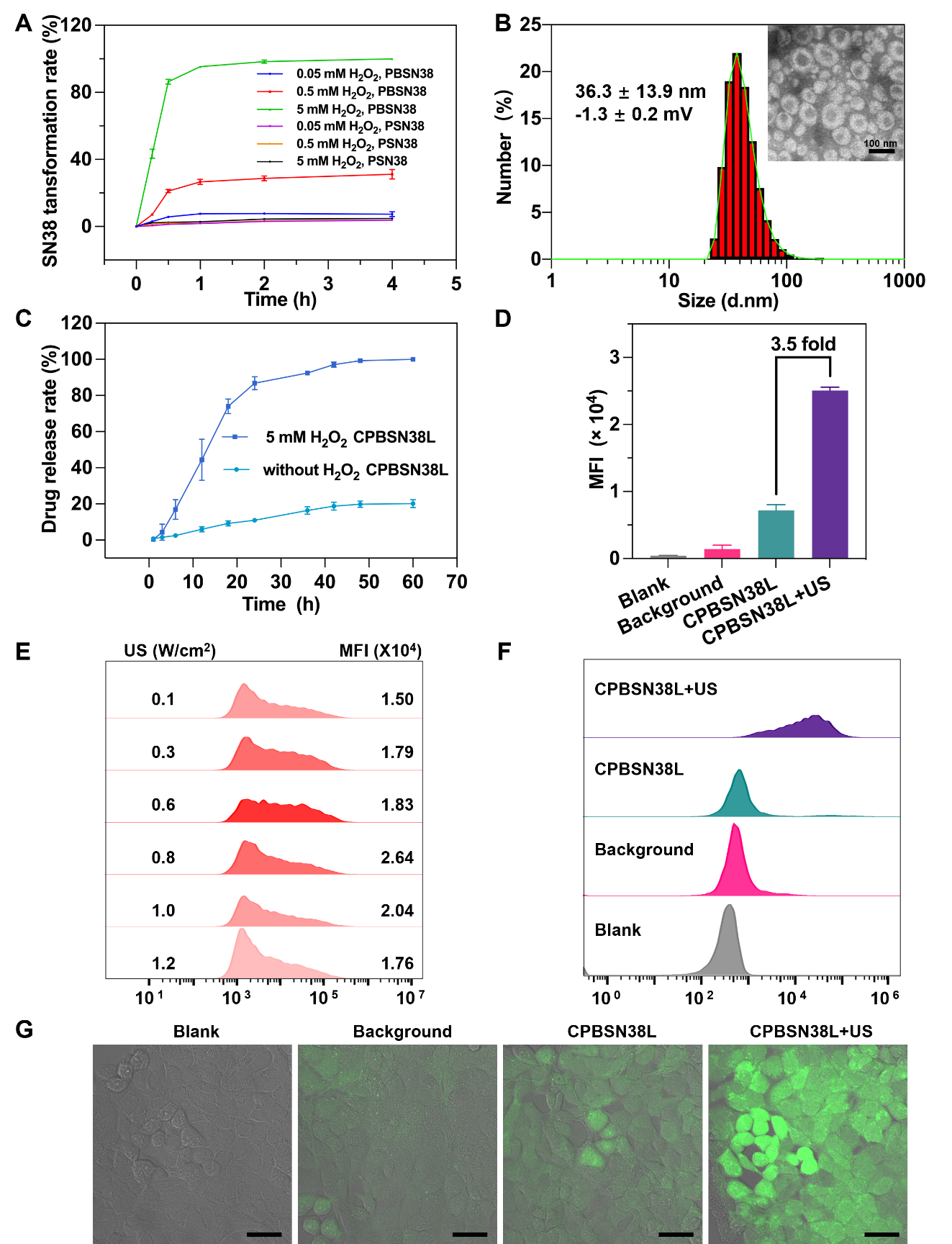


**Fig. 1** Characterizations of the prodrugs and prodrugs-loaded liposome. **A** Prodrugs transformation at different incubation times and concentrations of H_2_O_2_. The PBSN38 and PSN38 were separately incubated with 0.05~5 mM H_2_O_2_ solution with the prodrug and H_2_O_2_ at a molar ratio of 1%. **B** The size and zeta potential of CPBSN38L measured by dynamic light scattering (DLS). Morphology is shown as observed by transmission electron microscopy (TEM). Scale bar = 100 nm. **C** The kinetic of SN38 release from CPBSN38L in a PBS solution containing 10% FBS and 2% glycerol without and with H_2_O_2_ by using a dialysis bag at room temperature. **D** Quantitative results of the flow cytometry analysis of the intracellular ROS levels in MC38 colon adenocarcinoma cells treated with PBSN38L (blank group), PBSN38L + DCFHDA (background group), CPBSN38L + DCFHDA (CPBSN38L group), and CPBSN38L+US + DCFHDA (CPBSN38L+US group). **E** Flow cytometry assay of different acoustic intensity-induced ROS production of CPBSN38L. **F** Representative flow cytometry plots and **G** confocal laser scanning microscopy (CLSM) images of the intracellular ROS production of CPBSN38L. MC38 cells were incubated with CPBSN38L for 4 h and detected by DCFHDA staining (green). Scale bar = 50 µm. The data are presented as the mean ± SD (n = 3).

**Intracellular Efficient ROS Generation**

We subsequently monitored the efficiency of Ce6 to induce intracellular ROS generation in MC38 murine colon cancer cells. 2′-7′-Dichlorodihydrofluorescein diacetate (DCFHDA), a membrane-permeable precursor of dichlorofluorescein (DCF), was used as an intracellular ROS-sensitive fluorescent probe. When oxidized by ROS, this probe yields the highly fluorescent product DCF.^[^[^37^](#_ENREF_37)^]^ Then, the impermeable DCF accumulated in the cells was monitored through increased green fluorescence under excitation at 485 nm, which reflected the amount of ROS produced in tumor cells. After incubation with CPBSN38L for 6 h, the cells were stained with DCFHDA and irradiated with low to high acoustic intensities of US (US intensity: 0, 0.1, 0.3, 0.6, 0.8, 1.0, 1.2 W/cm^2^; transducer frequency of 3 MHz with a 50% duty cycle for 5 min). The fluorescence intensity of ROS production was detected and quantitated through flow cytometry (Fig. 1D–F and Fig. S6, Supporting Information). The combination of CPBSN38L and 0.8 W/cm^2^ US irradiation significantly increased ROS concentration in MC38 cells compared with those treated with CPBSN38L alone or with lower US irradiation (Fig. 1D and 1F). Interestingly, the fluorescence intensity of intracellular ROS decreased after treatment with CPBSN38L and greater-than 0.8 W/cm^2^ US irradiation, which might be a result of sonodynamic therapy-induced cell rupture under the stronger US intensity. To ensure more intuitive and visual ROS generation in cells, confocal laser scanning microscopy (CLSM) was performed. CLSM images verified that DCF fluorescence was very weak in the CPBSN38L group without US irradiation. By contrast, the green fluorescing signals were considerably stronger in the CPBSN38L and 0.8 W/cm^2^ US irradiation group, indicating considerably higher intracellular ROS levels (Fig. 1G). Considering that Ce6 under 0.8 W/cm^2^ US irradiation largely induced considerable ROS production, US irradiation at 0.8 W/cm^2^ was selected as the optimal acoustic intensity for the subsequent experiments.

**Cellular Uptake and Subcellular Distribution**

The cellular uptake and subcellular distribution of CPBSN38L in MC38 cells were then investigated (Fig. 2A–D). The cellular uptake of Cy5-labeled liposome was quantitatively evaluated through flow cytometry analysis at different time points within 24 h. The cell fluorescence intensity cells gradually increased with an increase in the incubation time (Fig. 2C and 2D). The cells exhibited the fastest uptake within 6 h, with the internalization of more than 85% of the liposomes (Fig. S7, Supporting Information), and so, US irradiation was performed at 6 h after liposome incubation in the cell culture. The intracellular distribution of CPBSN38L increased when the incubation time was prolonged (Fig. 2A and 2B), which corresponded with the trends of cellular uptake. CPBSN38L was distributed not only into the lysosomes but also into the mitochondria that produce ROS after 6 h incubation, possibly inducing the synergistic effect of chemotherapy and sonodynamic therapy.

**Biocompatibility and Stability**

Hemolysis was subsequently performed to establish the biocompatibility of liposomes in circulation. CPBSN38L and PBSN38L both caused hemolysis with an increase in the concentration, but the values were less than 5% within the 10 mg/mL concentrations (Fig. S8A and S8B, Supporting Information). CPBSN38L and PBSN38L were stable for 3 weeks in PBS or PBS containing 10% FBS, and no precipitates were formed within 1 week of storage at room temperature (Fig. S8C, Supporting Information). Together, these data suggest that liposomes are stable and biocompatibility for further in vivo use.


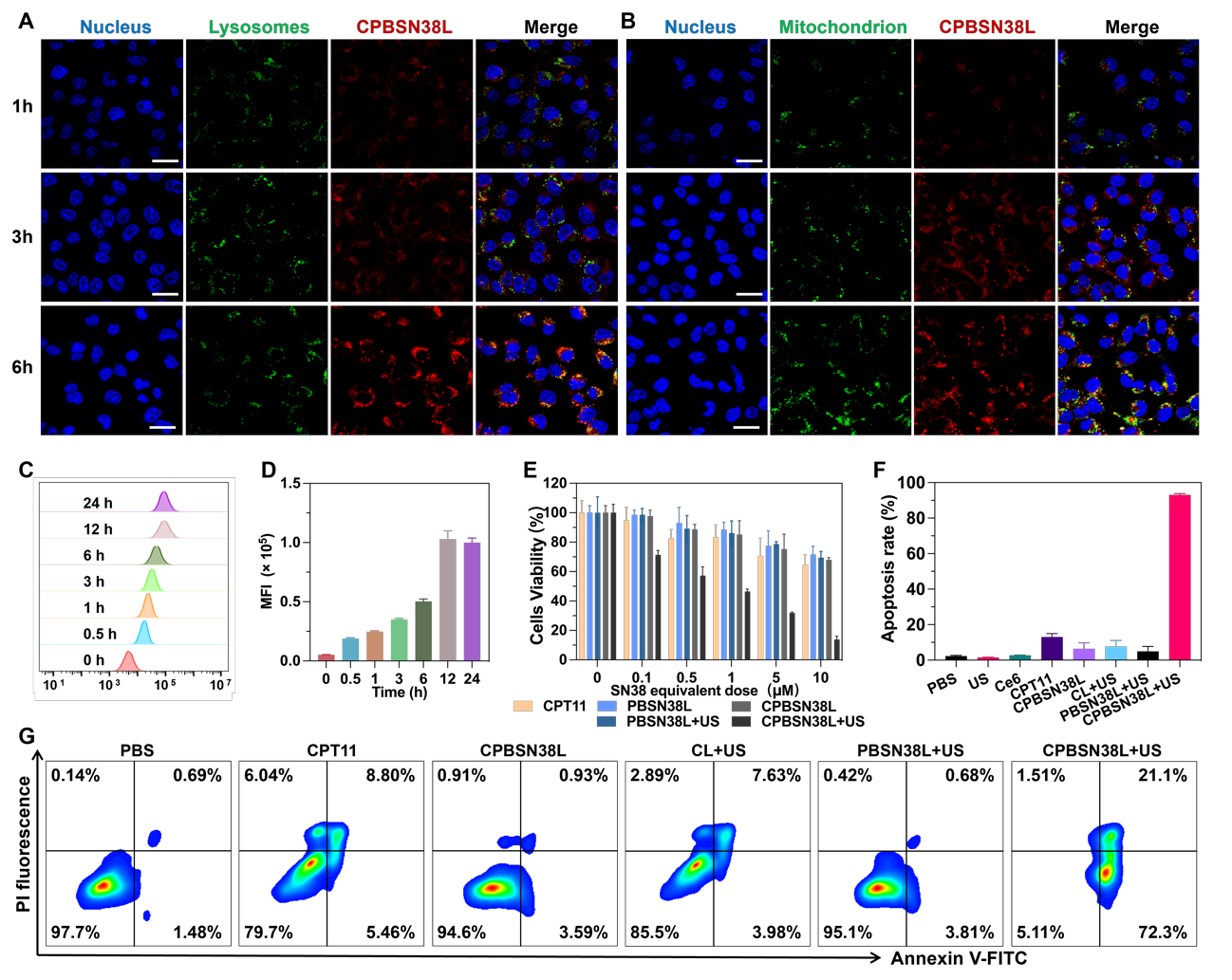


**Fig. 2** Cellular uptake, subcellular distribution, and cytotoxicity. **A, B** Subcellular distribution of the liposomes in tumor cells. Nucleus, lysosomes, and mitochondrion were stained with Hoechst 33342 (blue), LysoTracker Green (green), and MitoTracker Green (green), respectively. Scale bar = 50 µm. **C** The cellular uptake of Cy5-labeled liposomes in MC38 cells was examined by flow cytometry. **D** Quantification of fluorescence intensity of the cellular uptake in a time-dependent manner. **E** Cell viability was measured by CCK-8 assay after treatment with CPT11, PBSN38L, and CPBSN38L in MC38 cells without or with ultrasound irradiation under different SN38-equivalent concentrations. The cells were incubated with drugs for 6 h, followed by US irradiation under the intensity of 0.8 W/cm^2^, 3 MHz, 50% of the duty cycle for 5 min, followed by incubation for 42 h. **F** Flow cytometry analysis of cell apoptosis after different treatments, and the **G** representative flow cytometry dot plot in each group. The SN38-equivalent concentration is 10 µM.

**In Vitro Cytotoxicity**

The in vitro cytotoxicity of the prodrug and liposomes was then investigated. After treatment with different SN38-equivalent concentrations without US irradiation, the free drug SN38 exhibited the highest cytotoxicity against MC38 cells. The cell viability rate of the SN38 group was less than 50%, whereas that of the PBSN38 and PSN38 groups was 62.5% and 78.9% at as high as the SN38-equivalent concentration of 5 µM, respectively (Fig. S9A, Supporting Information). This indicated that the prodrug alone cannot induce obvious cell death. At the same time, the cytotoxicity resulting from sonodynamic therapy was investigated. The cells were treated with different intensities of US (0~1.4 W/cm^2^) and 15 µM of the Ce6-equivalent concentration. No significant changes in cell viability were observed with an increase in acoustic intensities from 1.0 to 1.4 W/cm^2^, confirming that the ROS production level was far from the lethal dosage (Fig. S9B, Supporting Information). The synergistic activity of ROS generation and prodrug activation was then tested using cell counting kit-8 (CCK-8). Compared with other groups with or without US irradiation, CPBSN38L+US led to a significant increase in cytotoxicity against MC38 cells (Fig. 2E). Flow cytometric analysis was performed to simultaneously analyze cell apoptosis after Annexin-V-FITC/PI staining (Fig. 2F and 2G). The CPBSN38L alone-treated group exhibited slight tumor cell-killing ability with an apoptosis rate of less than 5%, whereas the combination of CPBSN38L and US irradiation led to an excellent improvement in cell apoptosis, exhibiting an apoptosis rate of more than 90%; and these results corresponded with those of the cytotoxicity experiment. The results thus collectively proved the sonosensitizer Ce6, along with US irradiation, produces intracellular ROS to trigger PBSN38 activation and induce cell apoptosis. This combination may be used to treat solid tumors in a spatially precise manner while avoiding the potential damage to normal tissues.

**In Vivo Antitumor Efficacy in Murine Colorectal Cancer Model**

The in vivo therapeutic efficacy of CPBSN38L at the SN38-equivalent dose of 5 mg/kg was compared with that of CPT11, CPBSN38L, CL+US, PBSN38L+US, and PBS in an MC38 tumor model (Fig. 3A–G). Once the tumor volume was approximately 70~90 mm^3^, the mice were randomized into six groups, and the formulations were intravenously administered. At 6 h after injection, the tumor region was irradiated with the US (3 MHz, 50% duty cycle) for 5 min at an acoustic intensity of 0.8 W/cm^2^. This selective irradiation could elevate the tumor ROS level due to Ce6 being co-assembled in liposomes, which triggered prodrug activation. Each treatment was performed every 3 days for a total of 5 times. On day 21, the tumors were excised, photographed (Fig. 3E), and weighed (Fig. 3D). The tumors grew rapidly in the control group. Administration of CPBSN38L, followed by US irradiation, had a remarkable tumor-killing effect (Fig. 3B and 3E). Meanwhile, the efficiency of liposomes without US irradiation or the sonosensitizer was limited. Compared with PBS, the inhibition rate of tumor growth (IRT) for CPBSN38L+US was 92.1%, which was markedly higher than the 57.6% observed for CPT11, 60.3% observed for CPBSN38L, and 49.7% for PBSN38L+US (Fig. 3D). Cancer cells exhibit elevated ROS levels because of oncogenic stimulation, mitochondrial malfunction, and metabolic aberration. Although cancer cells exhibit higher ROS levels than normal cells by an order of magnitude, endogenous ROS levels may not be sufficient to induce complete drug release because of the balancing ability of cellular redox status and tumor heterogeneity. By introducing the SDT effect in the drug release system to provoke active pharmaceutical ingredient precision released, in addition to a synergistic therapeutic effect with chemotherapeutic drugs on direct tumor-killing. As noted previously, such cascade activating amplified strategy could minimize the nonspecific targeting of normal tissues and organs by agents. Meanwhile, no significant loss in body weight was observed in each liposome group, but a transient trend of decreasing body weight was observed in the CPT11 group during the treatment cycle (Fig. 3C). This decrease may be attributable to the effect of the chemodrug on the gastrointestinal tract, which resulted in decreased appetite and nutrient absorption from the gut.

CPBSN38L biodistribution in vivo and ex vivo was also observed at 12 h after injection in MC38-Luci tumor-bearing mice (Fig. S10 and S11, Supporting Information). CPBSN38L preferentially accumulated in the liver and tumor than in other normal tissues. Liposomes significantly accumulate in the liver because of the properties of the liver and spleen enriched with reticuloendothelial cells and clearance of the mononuclear phagocytic system, as observed with the long-circulating liposomes having a diameter of 70~200 nm. As described previously, the prodrug may not be activated in normal tissues, including the liver, without US irradiation of the drug delivery system.

Serum collected from the mice following different treatments were used to determine liver function [based on the levels of alanine aminotransferase (ALT) and aspartate aminotransferase (AST)] and kidney function [(based on the levels of blood urea nitrogen (BUN) and creatinine (CREA)] to assess hepatic and renal toxicity in vivo, respectively. No significant differences were observed in serum ALT, AST, BUN, and CREA levels in the liposomes group, and all levels were in the normal range relative to the control group. By contrast, serum ALT and AST levels in the CPT11 group were higher than those in the control group, which suggested that chemotherapy-related toxic side effects may cause impairment of liver function (Fig. 3F). These results revealed that liposomes caused no major toxicities to the liver and kidney, suggesting their excellent biocompatibility and biosafety.

The histological study was conducted through the examination of hematoxylin and eosin (H&E)-stained tumor tissue sections (Fig. 3G). Tumor cells in the PBS control group existed in fullness and a tightly packed state, while the cells treated with CPBSN38L+US were swollen and exhibited severe nuclear shrinkage and fragmentation, which are typical morphological characteristics of apoptosis. These changes were also observed in the CPT11, CPBSN38L, and PBSN38L+US groups, but to a considerably lesser extent. Furthermore, immunofluorescence staining of Ki67 and terminal deoxynucleotidyl transferase-mediated 2'-deoxyuridine 5'-triphosphate nick end labeling (TUNEL) staining of tumor tissues was performed to investigate the effects of different treatments on the inhibition of proliferation and the induction of apoptosis. Compared with other groups, the CPBSN38L+US group exhibited significantly reduced Ki67-positive cells (as indicated through red fluorescence) (Fig. 3G), indicating that tumor cell growth was effectively inhibited. Moreover, TUNEL staining, which allows in situ detection of DNA damage (DNA fragmentation), was applied to investigate drug-induced cell apoptosis. The TUNEL assay results revealed more apoptotic cells (as indicated through green fluorescence) in the tumors of the CPBSN38L+US group than of the other groups (Fig. 3G). The outstanding therapeutic effect of the CPBSN38L+US in vivo against colorectal tumors correlated well with the in vitro cytotoxicity data. This proved that the cascade amplified drug release system with US-controlled ROS generation capability has the potential to significantly increase prodrug activation selectivity and tumor therapeutic efficacy.


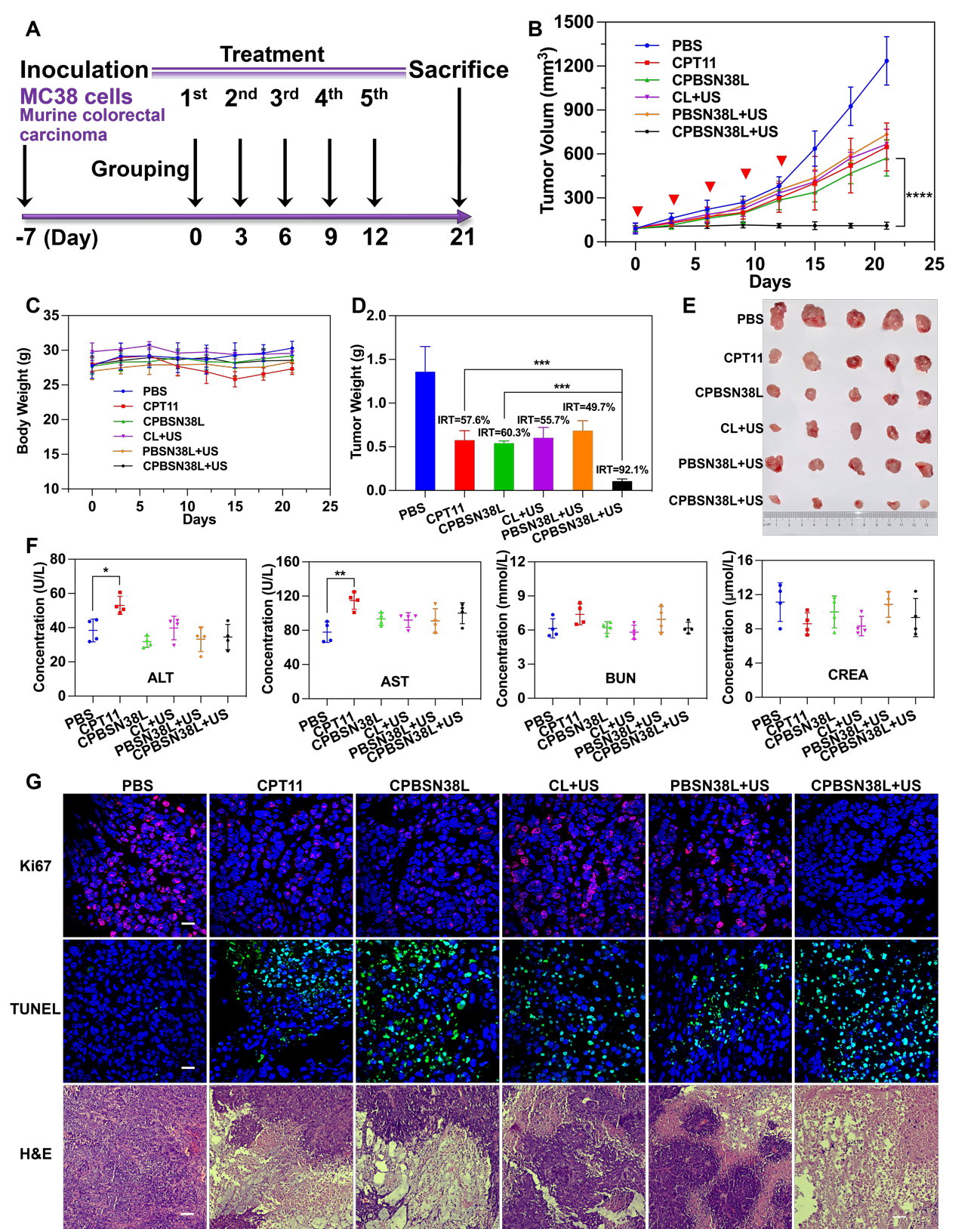


**Fig. 3** In vivo antitumor efficacy in a murine colorectal cancer model. The MC38 tumor bearing-mice were intravenously injected the formulations at SN38-equivalent dose of 5 mg/kg and then treated without or with US irradiation (0.8 W/cm^2^, 3 MHz, 50% of duty cycle, 5 min) at 6 h after intravenous injection. **A** The experimental timeline and tumor treatment schedule in murine colorectal tumor model. **B** The changes in the tumor volume during the treatment. **C** The changes in body weight in each group. **D** The inhibition rates of tumor growth (IRT) and tumor weights. **E** The photograph of resected MC38 tumors in each group. **F** Liver-related serum biochemical analysis of ALT and AST and the kidney function indicators of BUN and CREA. **G** Representative images of the Ki67, TUNEL, and H&E staining of the tumor slices. Scale bar = 50 µm (Ki67 and TUNEL staining) or 100 µm (H&E staining). Data were expressed as the mean ± SD (n = 5, ^***^*p* < 0.001, ^****^*p* < 0.0001).

**In Vivo Antitumor Efficacy and Side-effects Evaluation of the Liposome in Hepatocellular Carcinoma**

CPT11 and topotecan (TPT) are FDA-approved camptothecin (CPT) analogs for cancer treatment, especially in refractory and metastatic cancer. However, the serious side effects associated with these analogs have usually limited their clinical application. Using another highly malignant tumor of Huh7 human hepatoma, we evaluated the in vivo antitumor activity and treatment-associated toxicity of CPBSN38L versus CPT11 and TPT. The therapeutic effect was consistent with that observed in the colorectal tumor model. Tumor growth was remarkably inhibited in the CPBSN38L-treated mice compared with the CPT11- or TPT- and PBSN38L-treated mice, as shown in Fig. 4A–C and Fig S12, Supporting Information. In addition, no significant loss in body weight was observed in each liposome group compared with the CPT11 and TPT groups, in which body weights decreased to a different extent during treatment (Fig. 4D). Myelosuppression and enteritis are common side effects of CPT analogs. These side effects severely affect patient compliance and chemotherapy efficacy. On day 21, a routine blood examination of each mouse group was performed to evaluate chemotherapy-induced myelosuppression, as shown in Fig. 4E–K. The leukocyte count decreased significantly after CPT11 and TPT treatments than after PBS treatment (Fig. 4E), which is evidence of myelosuppression and the decline of hemoglobin levels in the CPT11 and TPT groups (Fig. 4G) often predicted more severe myelosuppressive. Platelet counts were also elevated in the CPT11 and TPT groups (Fig. 4F), which might contribute to hypercoagulability and are associated with a worse outcome in cancer patients. The elevated neutrophil levels (Fig. 4I) and a decline in the absolute number of lymphocytes (Fig. 4J) suggested the presence of systemic inflammation, which may also be related to enteritis. Chemotherapy-induced monocytopenia (Fig. 4K) was also in line with myelosuppression in the CPT11 and TPT groups. Furthermore, a decrease in leukocytes and an imbalance of the proportion of white blood cells may cause disorders of immune factors, leading to reduced immune function, which could make the patients vulnerable and result in treatment termination due to side effects. Moreover, hepatorenal function impairment in the TPT group and liver function damage in the CPT11 group were directly correlated with patient prognosis (Fig. 4L).

A typical pathology of intestinal inflammation was observed in the CPT11 and TPT groups (Fig. 4M). Villous denudation and crypt atrophy, the evidence of mucosal architecture destruction, were observed in the CPT11 and TPT groups. In addition, a disrupted surface epithelium and inflammatory cell infiltration in the villous lamina propria were observed in the TPT group. In the PBS and nanoliposome groups, the intestinal mucosa was intact, and the villi of their small intestine were well-arranged. Furthermore, the TUNEL assay was performed on intestinal sections to observe the extent of apoptosis in different groups. More apoptotic cells (as indicated through green fluorescence) were observed in the surface epithelium of the CPT11 and TPT groups than in the other groups (Fig. 4M), which further confirmed CPT11 and TPT that post-chemotherapy enteritis causes marked disruption of the intestinal tissue. In parallel, the difference between CPBSN38L and PBS was not obvious through these indices. No significant pathological abnormalities of main organs including hearts, livers, spleens, lungs, kidneys were observed in treated groups compared to the control group (Fig. S13, Supporting Information), which also indicate that all samples have no obvious chemotherapy induced damage in these normal tissues. The experimental results of CPBSN38L+US confirm that they have the targeted ability to activate therapeutic agents in the tumor site and achieve efficient anticancer treatment to fulfill precision therapy without side effects.


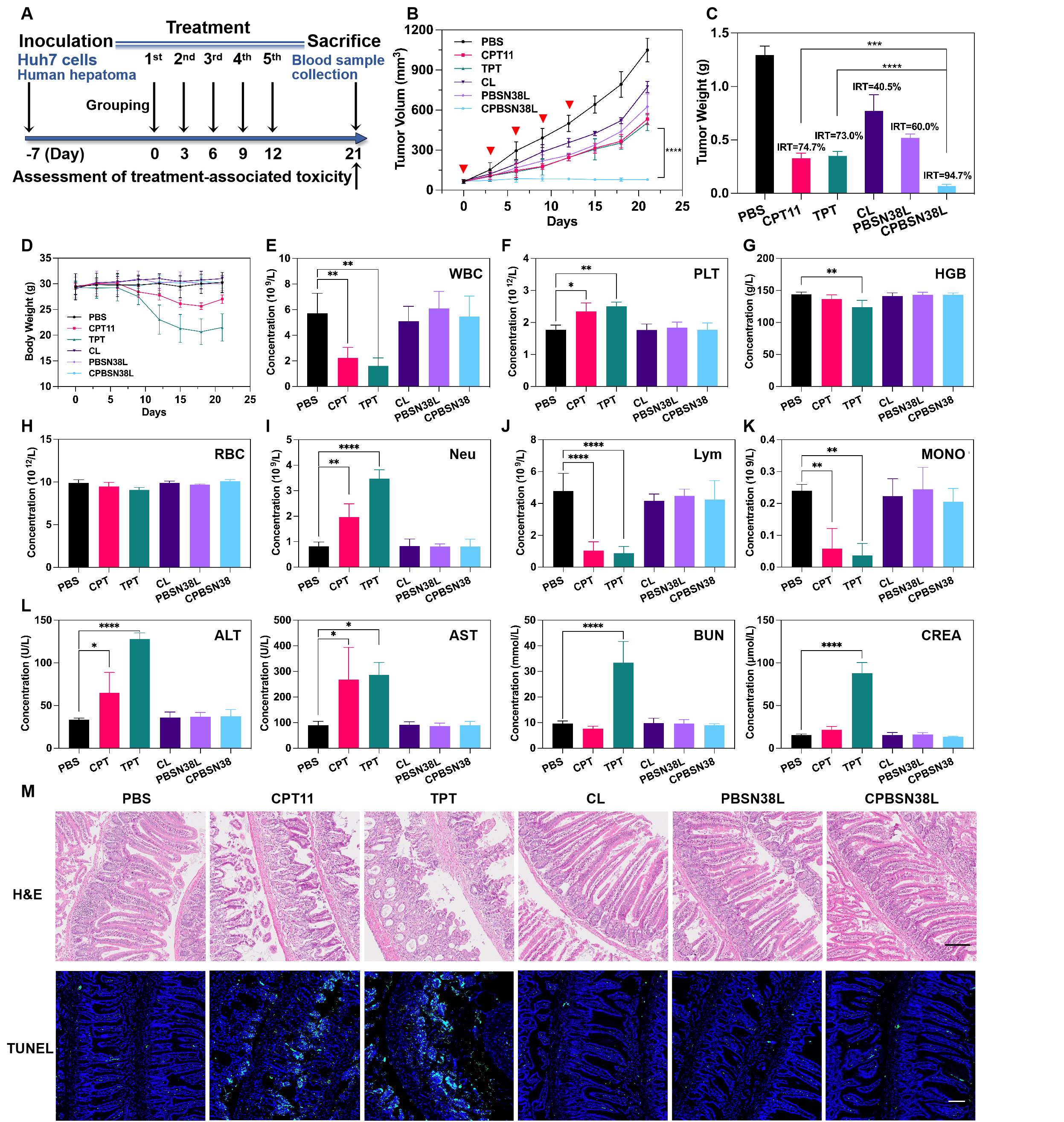


**Fig. 4** In vivo antitumor efficacy and side-effects evaluation of the liposome in hepatocellular carcinoma. The Huh7 tumor-bearing mice were intravenously injected with the formulations at an SN38-equivalent dose of 5 mg/kg and treated with US irradiation (0.8 W/cm^2^, 3 MHz, 50% of duty cycle, 5 min) at 6 h after intravenous injection in each group. The treatment was performed every 3 days for a total of 5 times, as indicated by red arrows. **A** The experimental timeline and tumor treatment schedule in this study. **B** The changes in the tumor volume during the treatment. **C** The inhibition rates of tumor growth (IRT) and tumor weights. **D** The changes in body weight in each group. **E–K** The blood cells and components of leukocytes, blood platelets, erythrocytes, neutrophils, monocytes, lymphocytes, and hemoglobin were evaluated in each peripheral blood sample. **L** Liver-related serum biochemical analysis of ALT and AST, and the kidney function indicators of BUN and CREA. **M** Representative images of the H&E staining and TUNEL assay staining of intestinal cross-sections in each group. Scale bar = 100 µm. Data were expressed as the mean ± SD (n = 5, ^***^*p* < 0.001, ^****^*p* < 0.0001).

**Conclusion**

In summary, a US-controlled prodrug activation platform was successfully fabricated by loading the ROS-responsive prodrug in sonosensitizer-decorated liposomes. We demonstrated the feasibility and effectiveness of the strategy of leaving the off-targeted drugs inactive but activating the tumor-distributed drugs for cancer-targeting therapy in a tumor microenvironment-independent manner. In this strategy, US irradiation was used to induce massive intracellular ROS production and initiate the cascade of amplified ROS-responsive PBSN38 activation to release active SN38 for inducing cell apoptosis. The designated CPBSN38L with US irradiation exhibited superior anticancer activity in multiple solid tumor models with no chemotherapy-induced side effects. The US-controlled activable liposomes are practical and efficient carriers for cancer targeting therapy in a side effect-evitable, universal, and tumor microenvironment-independent manner.

**Abbreviations**

**ALT**: alanine aminotransferase; **AST**: aspartate aminotransferase; **BUN**: blood urea nitrogen; **CCK-8**: cell counting kit-8; **Ce6**: chlorin e6; **CHEMS**: cholesteryl hemisuccinate; **CLSM**: confocal laser scanning microscopy; **CL**: Ce6-modified liposome; **CPBSN38L**: Ce6-modified and PBSN38-loaded liposome; **CPT**: camptothecin; **CPT11**: Irinotecan; **CREA**: creatinine; **Cy5**: cyanine-5; **DCF**: dichlorofluorescein; **DCFHDA**: 2′-7′-Dichlorodihydrofluorescein diacetate; **DLS**: dynamic light scattering; **DMEM**: Dulbecco's modified Eagle's medium; **DOPE**: 1,2-dioleoyl-sn-glycero-3-phosphoethanolamine; **DSPE-PEG**: 1,2-distearyl-sn-glycerol-3-phosphoethanolamine-polyethylene glycol; **dUTP**: 2'-deoxyuridine 5'-triphosphate; **EPR**: enhanced permeability and retention; **ER**: endoplasmic reticulum; **FBS**: fetal bovine serum; **GSH**: glutathione; **HGB**: hemoglobin; **H&E**: hematoxylin and eosin; **^1^H NMR**: proton nuclear magnetic resonance; **H_2_O_2_**: hydrogen peroxide; **HPLC**: high-performance liquid chromatography; **IRT**: inhibition rate of tumor growth; **Lym**: lymphocyte; **MALDI-TOF-MS**: matrix-assisted laser desorption/ionization time of flight mass spectrometry; **MFI**: mean fluorescence intensity; **MONO**: monocyte; **NAD(P)H**: nicotinamide adenine dinucleotide phosphate; **NEU**: Neutrophil; **NQO1**: quinone oxidoreductase-1; **O2^•–^**: superoxide anion; **^1^O_2_**: singlet oxygen; **^•^OH**: hydroxyl radical; **PBS**: phosphate-buffered solution; **PBSN38**: phenylboronic acid pinacol ester-conjugated SN38; **PBSN38L**: PBSN38-loaded liposome; **CPBSN38L^Cy5^**: Cy5-labeled CPBSN38L; **PDI**: polydispersity index; **PLT**: blood platelet; **PSN38**: phenylcarbinol-conjugated SN38; **RBC**: erythrocyte; **ROS**: reactive-oxygen-species; **SDT**: Sonodynamic therapy; **SN38**: 7-ethyl-10-hydroxycamptothecin; **TEM**: transmission electron microscope; **TdT**: terminal deoxynucleotidyl transferase; **TPT**: Topotecan; **TUNEL**: TdT-mediated dUTP nick end labeling; **US**: ultrasound; **WBC**: leukocyte.

**ASSOCIATED CONTENT**

**Supporting Information**

The online version contains supplementary material available at XXX.

**Additional file 1:** Additional experimental details and data, including: materials, synthesis of the prodrugs, the ^1^H-NMR spectrum, and the MALDI-TOF-MS spectrum, the HPLC spectrum, the cellular uptake, intracellular ROS assessments, in vitro hemolysis, cell viability tests, in vivo biodistribution, histological examination and immunofluorescent analysis of tumor tissues, assessment of treatment-associated toxicity and statistical analysis. **Scheme S1.** Synthesis of the prodrugs: **A** phenylboronic acid pinacol ester-conjugated SN38 (PBSN38), and **B** phenylcarbinol-conjugated SN38 (PSN38), and their ROS-responsive activation. **Figure S1.** The ^1^H-NMR spectrum of PBSN38 in CDCl3. **Figure S2.** The MALDI-TOF-MS spectrum of PBSN38. **Figure S3.** The ^1^H-NMR spectrum of PSN38 in CDCl3. **Figure S4.** The MALDI-TOF-MS spectrum of PSN38. **Figure S5.** Drug activation of PBSN38 and PSN38 were monitored by tracking the SN38 releasing. **A** PBSN38 (0.05 mM) was incubated with different concentrations of H_2_O_2_ in methanol solution at 37^o^C for 2 h. The samples of PBSN38 were analyzed using the Agilent ZORBAX Eclipse XDB-C18 column (75 × 4.6 mm, 3.5 µm) at 40^o^C in Agilent-1100. The mobile phase was eluted at 1.0 mL/min and consisted of acetonitrile/H_2_O (35:65, v/v). The detector was set at 254 nm. **B** PSN38 (0.05 mM) was incubated with different concentrations of H_2_O_2_ in acetonitrile and methanol mixture solution at 37^o^C for 2 h. The samples of PSN38 were analyzed using the mobile phase of methanol/H_2_O (30:70, v/v). **Figure S6.** Quantitative results of the flow cytometry analysis of the intracellular ROS levels in MC38 colon adenocarcinoma cells treated with CPBSN38L under different acoustic intensity. **Figure S7.** The flow cytometry analysis of cellular uptake rate of CPBSN38L in different time points. **Figure S8.** In vitro hemolysis was performed using mouse red blood cells after incubation with the liposomes. **A** Photographs of RBC suspensions after 2 h incubation in a shaker at 37 °C with PBSN38L and CPBSN38L at the lipid concentration in the range of 0.1-20 mg/mL. **B** Hemolysis rate of the liposomes at different concentrations. The saline was set as negative (0% hemolysis) and the 1% Triton X-100 was set as positive (100% hemolysis). **C** Photographs of *(1)* CPBSN38L and *(2)* PBSN38L in PBS containing (a) 0% or (b) 10% FBS at room temperature for one month. **Figure S9.** In vitro cell viability. **A** The cell viability of MC38 was detected by CCK-8 kit after incubation with free drug of SN38, or the prodrug of PBSN38 and PSN38 without US irradiation. **B** The cell viability of MC38 was tested after incubation with 15 µM Ce6 and treatment with US irradiation (US parameter: 3 MHz, 50% of duty cycle for 5 min). **Figure S10.** In vivo biodistribution of CPBSN38L. Representative bioluminescence images and fluorescence images in mice whole body or the excised organs (T: tumor, H: heart, L: liver, S: spleen, Lu: lung, K: kidney, I: intestines). **Figure S11.** The fluorescence intensity of CPBSN38L in different organs and tissues were quantified with the unit of radiant efficiency using the Living Image^®^-4.5 software. **Figure S12.** The photograph of resected Huh7 tumors in different groups. **Figure S13.** Representative images of the H&E staining of main organ sections in each group. All groups were treated with US irradiation (0.8 W/cm^2^, 3 MHz, 50% of duty cycle, 5 min) at 6 h after intravenous injection of drugs. Scale bar = 100 µm.

**Acknowledgements**

Not applicable.

**Corresponding Authors**

E-mail: huangpintong@zju.edu.cn, wangguowei@zju.edu.cn

**ORCID**

Guowei Wang: 0000-0003-1369-8738

Pintong Huang: 0000-0003-0747-5765

**Authors’ contributions**

PH and GW designed the study, participated in the supervision and coordination of the study; YJ, HC, TL and CZ conceived and designed the experiments; YJ, TL and CZ performed most of the experiments; YJ and HC analyzed the data; PH, GW and YJ contributed to the writing, review and revision of the manuscript. All authors were involved in the theoretical discussion and performing of the experiments. All authors read and approved the final manuscript.

**Funding**

This work is financially supported by the National Natural Science Foundation of China (No. 82030048, 82230069, 82371967, 82102191, 82001818), Key Research and Development Program of Zhejiang Province (No. 2019C03077), Natural Science Foundation of Zhejiang Province (No. LQ20H180011, and LQ21H180007).

**Data Availability**

All data generated or analyzed during this study are included in this published article.

**DECLARATIONS**

**Ethics approval and consent to participate**

All experiments involving animals (License No. IACUC-20200611-05) were performed with the approval of and in accordance with the guidelines of the Institutional Animal Ethics. Committee of Laboratory Animal Center of Zhejiang Chinese Medical University.

**Consent for publication**

All authors of this study agreed to publish.

**Competing interests**

The authors declare that they have no competing interests.

**Author details**

^1^Department of Ultrasound in Medicine, The Second Affiliated Hospital of Zhejiang University School of Medicine, Zhejiang University, Hangzhou 310009, China. ^2^Research Center of Ultrasound in Medicine and Biomedical Engineering, The Second Affiliated Hospital of Zhejiang University School of Medicine, Zhejiang University, Hangzhou 310009, China. ^3^Research Center for Life Science and Human Health, Binjiang Institute of Zhejiang University, Hangzhou 310053, China.

[**REFERENCES**](app:ds:references)

1. Sung H, Ferlay J, Siegel RL, Laversanne M, Soerjomataram I, Jemal A, Bray F. Global cancer statistics 2020: GLOBOCAN estimates of incidence and mortality worldwide for 36 cancers in 185 countries. Ca-Cancer J Clin. 2021;71:209-49.

2. Huang AC, Zappasodi R. A decade of checkpoint blockade immunotherapy in melanoma: understanding the molecular basis for immune sensitivity and resistance. Nat Immunol. 2022;23:660-70.

3. Chowell D, Yoo SK, Valero C, Pastore A, Krishna C, Lee M, Hoen D, Shi H, Kelly DW, Patel N, et al. Improved prediction of immune checkpoint blockade efficacy across multiple cancer types. Nat Biotechnol. 2022;40:499-506.

4. Chamma H, Vila IK, Taffoni C, Turtoi A, Laguette N. Activation of STING in the pancreatic tumor microenvironment: A novel therapeutic opportunity. Cancer Lett. 2022;538:215694.

5. Leidner R, Sanjuan Silva N, Huang H, Sprott D, Zheng C, Shih Y-P, Leung A, Payne R, Sutcliffe K, Cramer J. Neoantigen T-cell receptor gene therapy in pancreatic cancer. N Engl J Med. 2022;386:2112-9.

6. Wolchok JD, Chiarion-Sileni V, Gonzalez R, Grob JJ, Rutkowski P, Lao CD, Cowey CL, Schadendorf D, Wagstaff J, Dummer R, et al. Long-Term Outcomes With Nivolumab Plus Ipilimumab or Nivolumab Alone Versus Ipilimumab in Patients With Advanced Melanoma. J Clin Oncol. 2022;40:127-37.

7. Mok TSK, Wu YL, Kudaba I, Kowalski DM, Cho BC, Turna HZ, Castro G, Jr., Srimuninnimit V, Laktionov KK, Bondarenko I, et al. Pembrolizumab versus chemotherapy for previously untreated, PD-L1-expressing, locally advanced or metastatic non-small-cell lung cancer (KEYNOTE-042): a randomised, open-label, controlled, phase 3 trial. Lancet. 2019;393:1819-30.

8. Junttila MR, De Sauvage FJ. Influence of tumour micro-environment heterogeneity on therapeutic response. Nature. 2013;501:346-54.

9. Quail DF, Joyce JA. Microenvironmental regulation of tumor progression and metastasis. Nat Med. 2013;19:1423-37.

10. Galluzzi L, Humeau J, Buque A, Zitvogel L, Kroemer G. Immunostimulation with chemotherapy in the era of immune checkpoint inhibitors. Nat Rev Clin Oncol. 2020;17:725-41.

11. Ang MJY, Chan SY, Goh Y-Y, Luo Z, Lau JW, Liu X. Emerging Strategies in Developing Multifunctional Nanomaterials for Cancer Nanotheranostics. Adv Drug Delivery Rev. 2021;178:113907.

12. Cook AB, Decuzzi P. Harnessing Endogenous Stimuli for Responsive Materials in Theranostics. ACS Nano. 2021;15:2068-98.

13. Tran S, DeGiovanni PJ, Piel B, Rai P. Cancer nanomedicine: a review of recent success in drug delivery. Clin Transl Med. 2017;6:44.

14. Tee JK, Yip LX, Tan ES, Santitewagun S, Prasath A, Ke PC, Ho HK, Leong DT. Nanoparticles' interactions with vasculature in diseases. Chem Soc Rev. 2019;48:5381-407.

15. Izci M, Maksoudian C, Manshian BB, Soenen SJ. The Use of Alternative Strategies for Enhanced Nanoparticle Delivery to Solid Tumors. Chem Rev. 2021;121:1746-803.

16. Parodi A, Kolesova EP, Voronina MV, Frolova AS, Kostyushev D, Trushina DB, Akasov R, Pallaeva T, Zamyatnin AA, Jr. Anticancer Nanotherapeutics in Clinical Trials: The Work behind Clinical Translation of Nanomedicine. Int J Mol Sci. 2022;23:13368.

17. Oser MG, Fonseca R, Chakraborty AA, Brough R, Spektor A, Jennings RB, Flaifel A, Novak JS, Gulati A, Buss E, et al. Cells Lacking the RB1 Tumor Suppressor Gene Are Hyperdependent on Aurora B Kinase for Survival. Cancer Discovery. 2019;9:230-47.

18. Salvati A, Pitek AS, Monopoli MP, Prapainop K, Bombelli FB, Hristov DR, Kelly PM, Aberg C, Mahon E, Dawson KA. Transferrin-functionalized nanoparticles lose their targeting capabilities when a biomolecule corona adsorbs on the surface. Nat Nanotechnol. 2013;8:137-43.

19. Cai R, Chen C. The Crown and the Scepter: Roles of the Protein Corona in Nanomedicine. Adv Mater. 2019;31:e1805740.

20. Wang GW, Jiang YF, Xu JJ, Shen JX, Lin T, Chen JF, Fei WD, Qin YT, Zhou ZX, Shen YQ, Huang PT. Unraveling the Plasma Protein Corona by Ultrasonic Cavitation Augments Active-Transporting of Liposome in Solid Tumor. Adv Mater. 2023;35:e2207271.

21. Manzari MT, Shamay Y, Kiguchi H, Rosen N, Scaltriti M, Heller DA. Targeted drug delivery strategies for precision medicines. Nat Rev Mater. 2021;6:351-70.

22. Ho WJ, Jaffee EM, Zheng L. The tumour microenvironment in pancreatic cancer - clinical challenges and opportunities. Nat Rev Clin Oncol. 2020;17:527-40.

23. Ouyang J, Tang Z, Farokhzad N, Kong N, Kim NY, Feng C, Blake S, Xiao Y, Liu C, Xie T, Tao W. Ultrasound mediated therapy: Recent progress and challenges in nanoscience. Nano Today. 2020;35:100949.

24. Son S, Kim JH, Wang X, Zhang C, Yoon SA, Shin J, Sharma A, Lee MH, Cheng L, Wu J, Kim JS. Multifunctional sonosensitizers in sonodynamic cancer therapy. Chem Soc Rev. 2020;49:3244-61.

25. Chen Y, Li Y, Du M, Yu J, Gao F, Yuan Z, Chen Z. Ultrasound Neuromodulation: Integrating Medicine and Engineering for Neurological Disease Treatment. BIO Integr. 2022;2:169-79.

26. Zhang C, Chen J, Song Y, Luo J, Jin P, Wang X, Xin L, Qiu F, Yao J, Wang G, Huang P. Ultrasound-Enhanced Reactive Oxygen Species Responsive Charge-Reversal Polymeric Nanocarriers for Efficient Pancreatic Cancer Gene Delivery. Acs Appl Mater Interfaces 2022;14:2587-96.

27. Wang G, Zhang C, Jiang Y, Song Y, Chen J, Sun Y, Li Q, Zhou Z, Shen Y, Huang P. Ultrasonic Cavitation-Assisted and Acid-Activated Transcytosis of Liposomes for Universal Active Tumor Penetration. Adv Funct Mater. 2021;31:2102786.

28. Shi Z, Wu J, Song Q, Gostl R, Herrmann A. Toward Drug Release Using Polymer Mechanochemical Disulfide Scission. J Am Chem Soc. 2020;142:14725-32.

29. Huo S, Zhao P, Shi Z, Zou M, Yang X, Warszawik E, Loznik M, Gostl R, Herrmann A. Mechanochemical bond scission for the activation of drugs. Nat Chem. 2021;13:131-9.

30. Wang G, Zhou Z, Zhao Z, Li Q, Wu Y, Yan S, Shen Y, Huang P. Enzyme-Triggered Transcytosis of Dendrimer-Drug Conjugate for Deep Penetration into Pancreatic Tumors. ACS Nano 2020;14:4890-904.

31. Si J, Zhao X, Gao S, Huang D, Sui M. Advances in delivery of Irinotecan (CPT-11) active metabolite 7-ethyl-10-hydroxycamptothecin. Int J Pharm. 2019;568:118499.

32. Antunes F, Cadenas E. Cellular titration of apoptosis with steady state concentrations of H_2_O_2_: Submicromolar levels of H_2_O_2_ induce apoptosis through Fenton chemistry independent of the cellular thiol state. Free Radical Biol Med. 2001;30:1008-18.

33. Liou GY, Storz P. Reactive oxygen species in cancer. Free Radical Res. 2010;44:479-96.

34. Hayes JD, Dinkova-Kostova AT, Tew KD. Oxidative Stress in Cancer. Cancer Cell. 2020;38:167-97.

35. Ong W, Yang Y, Cruciano AC, McCarley RL. Redox-triggered contents release from liposomes. J Am Chem Soc. 2008;130:14739-44.

36. Pezzoli D, Tallarita E, Rosini E, Candiani G. Characterization and Investigation of Redox-Sensitive Liposomes for Gene Delivery. Methods Mol Biol. 2016;1445:217-33.

37. Rota C, Chignell CF, Mason RP. Evidence for free radical formation during the oxidation of 2 '-7 '-dichlorofluorescin to the fluorescent dye 2 '-7 '-dichlorofluorescein by horseradish peroxidase: Possible implications for oxidative stress measurements. Free Radical Biol Med. 1999;27:873-81.
