# Supplementary material for: Ultrasound-activated prodrug-loaded liposome for efficient cancer targeting therapy without chemotherapy-induced side effects
Source: J Nanobiotechnology. 2024 Jan 3;22:2. doi: 10.1186/s12951-023-02195-5 (PMC10763105; doi:10.1186/s12951-023-02195-5)
Supplement: Supplementary file 2 — Supplementary Material 2: Supporting schemes and figures [file 12951_2023_2195_MOESM2_ESM.docx]

**Supporting Information**

**Ultrasound-Activated Prodrug-Loaded Liposome for Efficient Cancer Targeting Therapy without Chemotherapy-Induced Side Effects**

Yifan Jiang,^1, 2, †^ Hongjian Chen,^1, 3^^,^ ^†^ Tao Lin,^1, †^ Chao Zhang,^1, 2^ Jiaxin Shen,^1, 2^ Jifan Chen,^1, 2^ Yanan Zhao,^1^ Wen Xu,^1^ Guowei Wang, ^1, 2, *^ and Pintong Huang ^1, 2, 3, *^

^1^ Department of Ultrasound in Medicine, The Second Affiliated Hospital of Zhejiang University School of Medicine, Zhejiang University, Hangzhou 310009, China.

^2^ Research Center of Ultrasound in Medicine and Biomedical Engineering, The Second Affiliated Hospital of Zhejiang University School of Medicine, Zhejiang University, Hangzhou 310009, China.

^3^ Research Center for Life Science and Human Health, Binjiang Institute of Zhejiang University, Hangzhou 310053, China.

^†^ These authors contributed equally to this work.

^*^Corresponding author, E-mail: wangguowei@zju.edu.cn, and huangpintong@zju.edu.cn

**Experimental Section**

**Materials.** All chemical reagents other than indicated were purchased from Sigma-Aldrich Inc. or Aladdin Reagent Inc. 1,2-distearyl-sn-glycerol-3-phosphoethanolamine-polyethylene glycol-2000 (DSPE-PEG), 1,2-Dioleoyl-sn-glycero-3-phosphoethanolamine (DOPE), Chlorin e6 (Ce6)-conjugated DSPE-PEG (DSPE-PEG-Ce6), Cyanine5 (Cy5)-labeled DSPE-PEG (DSPE-PEG^Cy5^) were purchased from Xi’an Ruixi Biological Technology Co., Ltd. Cholesteryl hemisuccinate and irinotecan (CPT11) were purchased from Shanghai Yuanye Bio-Technology Co., Ltd. DMEM medium, fetal bovine serum (FBS) and 0.25% trypsin solution were purchased from GIBCO. The Cell Counting Kit-8 (CCK-8) proliferation assay kit and Reactive Oxygen Species Assay Kit were purchased from Beyotime Biotechnology. Hoechst 33342, LysoTracker® Green DND26 and MitoTracker^®^ Green (green) were purchased from Thermo Fisher Scientific Inc. TUNEL Apoptosis Assay Kit was purchased from Roche, BD Pharmingen. Ki67 antibody was purchased from Proteintech Group. Annexin V-FITC/PI Apoptosis Detection Kit was purchased from Solarbio Science & Technology Co., Ltd.

**Synthesis and characterization of the prodrugs.** The synthesis route of PBSN38 and PSN38 are shown in Supporting Information, **Scheme S1**. First, 4-(hydroxymethyl) phenylboronic acid pinacol ester (7.37 g, 31.5 mmol) was dissolved in dry dichloromethane (DCM, 50 mL) in a flame-dried 100-mL flask, followed by addition of Carbonyl diimidazole (CDI, 10.20 g, 62.9 mmol) and stirring for 30 min. The mixture was diluted in ethyl acetate (200 mL) and washed with H_2_O (3 × 10 mL). The organics were washed with brine (2 × 10 mL), dried over MgSO_4_, and concentrated in vacuo to give a pure white solid of CDI-activated pinacol boronic ester (7.9 g, approximately 75.6% yield). Next, CDI-activated pinacol boronic ester (0.98 g, 3 mmol), SN38 (1.57 g, 4 mmol) and dimethylaminopyridine (DMAP, 0.3 mmol, 37 mg) were dissolved in anhydrous DCM (50 mL) and stirred for 6 h at room temperature. The solvent was removed by vacuum evaporation and chromatographic separation (silica gel, DCM/ethyl acetate = 3:2, v/v). The final product was dried in a vacuum oven, yielding a yellowish white solid of PBSN38 (1.81 g, approximately 70.2% yield). Similarly, the control prodrug of PSN38 was synthesized by conjugating with the phenylcarbinol.

The prodrugs’ structures were verified by proton nuclear magnetic resonance (^1^H NMR) (Avance-600 NMR, Bruker) and confirmed by matrix-assisted laser desorption ionization time-of-flight mass spectrometry (MALDI-TOF-MS). For MALDI-TOF-MS analysis, the sample solution (1 µL, 1 mg/mL dissolved in methanol) was mixed with the matrix solution (1 µL, 10 mg/mL, 2,5-dihydroxybenzoic acid) on the stainless-steel probe and was dried at room temperature. The laser power was between 50-100 µJ. PBSN38 (Supporting Information, **Figure S1**): ^1^H NMR (600 MHz, CDCl_3_, *δ*): 8.20 (s, 2H), 7.71 (s, 3H), 7.49 (s, 2H), 7.32 (s, 1H), 6.49 (s, 1H), 5.37 (d, J = 62.2 Hz, 7H), 3.29 (s, 3H), 2.48 (s, 2.3H), 1.86 (s, 2H), 1.29 (s, 15H), 0.87 (s, 3H), and the molecular weight of [M+H]^+^ in MALDI-TOF-MS was 653.26 (Supporting Information, **Figure S2**). PSN38 (Supporting Information, **Figure S3**): ^1^H NMR (600 MHz, CDCl_3_, *δ*): 8.16 (s, 2H), 7.45 (d, J = 43.7 Hz, 4H), 7.32 (s, 1H), 6.49 (s, 1H), 5.49-5.26 (m, 6H), 3.29 (s, 5H), 2.48 (s, 2H), 1.86 (s, 2H), 1.26 (s, 3H), 0.87 (s, 3H), and the molecular weight of [M+H]^+^ in MALDI-TOF-MS was 527.18 (Supporting Information, **Figure S4**).

**ROS-responsive activation of the prodrugs.** The cleavage of pinacol boronic ester and hydrolysis of carbonic ester were determined by incubating PBSN38 or PSN38 (50 µg/mL) in 2 mL methanol solution with H_2_O_2_ at a variety of concentrations (0, 0.05, 0.5, 5 mM) at 37 °C. At predetermined time intervals, the 100 µL mixture was collected for further analysis via high-performance liquid chromatography (HPLC). The mobile phase was eluted at 1.0 mL/min and consisted of acetonitrile/H_2_O (35:65 for PBSN38, and 30:70 for PSN38, v/v). The detector was monitored at 254 nm. HPLC was performed using the Agilent ZORBAX Eclipse XDB-C18 column (75 × 4.6 mm, 3.5 µm) at 40 °C in Agilent-1100.

**Preparation and characterization of liposomes**. The liposomes were prepared using the method of transmembrane ammonium sulfate gradient with modifications.^[1, 2]^ Taking the Ce6-modified and PBSN38-loaded liposome (CPBSN38L) for example, the lipids of DSPE-PEG-Ce6 (3.0 mg), DOPE (3.0 mg), DSPE-PEG (1.0 mg), and CHEMS (1.0 mg) were mixed at a 1.5:1.5:1:1 mass ratio in 50% (w/v) ethanolic solution, and then added with 10 volumes of the solution of triethylammonium salts of sucrose octasulfate (0.5 mM, pH 6.0) at 50 °C. The lipids solution was extruded through the polycarbonate filter of 100 and 50 nm pore size (Avanti Mini-Extruder Set, Avanti Polar Lipid Inc). Unentrapped triethylammonium salts were removed by chromatography on a Sepharose CL-4B size exclusion column eluted with HEPES-buffered dextrose (5 mM HEPES, 5% dextrose, pH 6.0). PBSN38⋅HCl was then added to the liposomes at a mass ratio of 1.25 mg PBSN38 per10 mg lipids at 50 °C with mechanically agitated for 30 min, and the pH adjusted to 6.0. Unencapsulated PBSN38 was subsequently removed using a Sephadex G-75 column eluted with HEPES-buffered saline (5 mM HEPES, 100 mM NaCl, pH 6.0). The loading efficiency was determined in all liposomes by quantitating the prodrug using HPLC. The loading efficiency = mass of prodrug in liposome / mass of total (lipids + prodrug) × 100%.

Similarly, with everything else being the same as the CPBSN38L fabrication, the PBSN38-loaded liposome without the DSPE-PEG-Ce6 lipid (PBSN38L) was fabricated with lipid mixture consisting of DOPE (3.0 mg), DSPE-PEG (4.0 mg), and CHEMS (1.0 mg); and the Ce6-modified liposome without PBSN38-loading (CL) was fabricated with lipid mixture consisting of DSPE-PEG-Ce6 (3.0 mg), DOPE (3.0 mg), DSPE-PEG (1.0 mg), and CHEMS (1.0 mg) without the prodrugs. At the same time, the Cy5-labeled liposomes were prepared by adding Cy5-labeled DSPE-PEG (DSPE-PEG^Cy5^) (0.20 mg, approximately 2.5% mass ratio of the total lipids) into the prescription to instead of the equivalent DSPE-PEG as the method mentioned above.

**SN38 release experiment.** CPBSN38L prepared as described above were diluted in phosphate-buffered solution (5 mL, containing 1mg of PBSN38). The solution was sealed in a dialysis bag with a 3500 molecular weight cut-off and dialyzed in 195 mL of PBS containing 10% FBS and 2% glycerol with or without 0.05 mM H_2_O_2_. At certain time intervals (1, 3, 6, 12, 18, 24, 36, 42, 48 and 60 h), a 100 µL portion of the solution outside the bag was subjected to the HPLC analysis, as described above.

**Cell culture.** The murine colorectal carcinoma MC38 cells and the human liver cancer Huh7 cells were purchased from American Type Culture Collection (ATCC). The cells were cultured in Dulbecco's modified Eagle's medium (DMEM) supplemented with 10% fetal bovine serum and 1% penicillin/streptomycin, and were maintained in a 5% CO_2_ humidified atmosphere at 37 °C.

**Intracellular ROS assessments.** Intracellular ROS production was examined by 2′,7′-dichlorodihydrofluorescein diacetate (DCFHDA; 10 µM) as a probe using a reactive oxygen species assay kit. For flow cytometry analysis, MC38 cells were seeded into 12-well plates and incubated overnight. Following 6 h incubation with PBSN38L or CPBSN38L in culture medium, cells were washed in PBS and loaded with 10 µM oxidant-sensitive dye DCFHDA in serum-free medium for 20 min. Subsequently, the cells in CPBSN38L+US group were performed with US irradiation (US parameter: 3 MHz, 50% of duty cycle for 5 min) and immediately washed twice. Cells in each group were resuspended and transferred into the tube. The fluorescence intensity of intracellular DCF was quantified by a Beckman CytoFlex flow cytometer. The DCF fluorescence images were acquired through Nikon A1 confocal microscope. MC38 cells were seeded on 35 mm glass-bottom petri dishes at 3 × 10^5^ cells/mL, 1 mL per dish incubated for 24 h and then processed as described above until the completion of DCFHDA staining. After washing 3 times with cold PBS, intracellular ROS production images were obtained with a confocal laser scanning microscope (CLSM) at excitation wavelength of 488 nm and emission wavelength of 523 nm channel.

**Cellular uptake and Sub****cellular distribution.** MC38 cells were seeded at a density of 3 × 10^5^ cells per well in 12-well plates and cultured for 24 h. CPBSN38L^Cy5^ (DSPE-PEG-Cy5 accounts for 20% ratio of DSPE-PEG, 15 µM, 9 µg/mL) with equal fluorescence intensity was added and incubated at 37 °C for 0, 1, 3, 6, 12, 24 h. The cells were washed and harvested by 0.25% Trypsin-EDTA and further centrifuged at 1000 rpm for 5 min. After washed and resuspended, the intracellular fluorescence of each sample was detected by flow cytometry. Each sample collected 10,000 gated events and the data were analyzed using CellQuest Pro software. MC38 cells were cultured a density of 1 × 10^5^ cells on 35 mm glass-bottom petri dishes with 1 mL medium for 48 h. The medium was replaced with 1 mL fresh serum-free medium containing CPBSN38L and incubated at 37 °C for 1 h, 3 h and 6 h. Subsequently, cells were washed with PBS to remove liposomes. The cells were further stained with LysoTracker^®^ Green (0.2 µL per dish, 30 min) and Hoechest 33342 (2 drops per dish, 15 min) to label lysosomes and nuclei, whereas MitoTracker^®^ Green (0.2 µL per dish, 30 min) and Hoechest 33342 (2 drops per dish, 15 min) to visualize the spatial location of mitochondria, nuclei, and liposomes. The subcellular distribution images of liposomes were acquired with CLSM using 405, 488 and 640 nm wavelength channels, separately.

**Hemolysis and Stability test.** Fresh anticoagulated mouse whole blood was collected and then the erythrocytes were isolated by centrifugation at 1500 rpm for 10 min and washed 3 times with PBS (pH 7.4). The stock solution of the erythrocytes was prepared using PBS and adjusted to 1 × 10^8^ cells/mL. PBSN38L and CPBSN38L suspensions were added into centrifuge tubes and diluted with PBS to bring the final volume to 0.9 mL with varying concentrations. Next, 0.1 mL of erythrocytes solution was added to the different dilutions. After incubation in a 37 °C shaker at 60 rpm for 2 h, intact erythrocytes were obtained by centrifugation at 1500 rpm for 10 min. The absorbance of hemoglobin supernatant was measured at 540 nm. Hemolysis ratios were calculated using the following: equation Hemolysis ratio = (A_sample_-A_negative_) / (A_positive_-A_negative_) × 100%, where the PBS and 1% Triton solution were used as negative and positive control, respectively.

**Cytotoxicity and apoptosis test in vitro**. The cytotoxicity of prodrugs and liposomes was evaluated by CCK-8 kit on the MC38 cell lines. Briefly, cells were seeded in 96 well plates at a density of 5000 cells per well and 96 wells of each plate were subdivided into 10 groups (longitudinal six wells as a group) or 6 groups (square region of four wells as a group) in terms of the application of ultrasonic transducer. After incubated overnight, cells were exposed to different processes: incubation with various concentrations (nine serial dilutions of equivalent SN38 in six replicates) of free drug SN38, prodrugs PBSN38 and PSN38 without US irradiation, or treatment with different ultrasonic intensity of US irradiation (3MHz, 50% duty cycle, 5 min) after 6h incubation with 15 µM Ce6, or incubation with five serial dilutions of equivalent SN38 of PBSN38L, CPBSN38L and CPT11 for 6 h with or without US irradiation (US parameter: 3 MHz, 50% of duty cycle for 5 min), and then the samples were incubated in fresh medium for an additional 48 h. According to CCK-8 cell proliferation assay protocol, 10 µL of CCK-8 solution and 90 µL DMEM were added to each well, after 1 h, optical density was measured at 450 nm using a microplate spectrophotometer. Cell viability after different treatments was calculated as the percentage of the absorbance in untreated cells.

Apoptosis and cell death were assayed via FITC Annexin-V and PI double staining using Annexin V-FITC Apoptosis Detection Kit following the manufacturers protocol. MC38 cells were seeded into 12-well plates at 3 × 10^5^ cells per well and incubated overnight for subjected to various treatments (PBS, US, CL, CPT11, CPBSN38L, CL+US, PBSN38L+US, CPBSN38L+US). SN38-equivalent concentration is 10 µM and US irradiation for 5 min after 6 h dosing and further incubation for 48 h. After washed with PBS and collected by trypsin digestion, the cells were counted and stained with Annexin V-FITC and PI at room temperature in the dark followed by flow cytometry analysis. In the dot plot analyses, the first quadrant represented cells were in healthy condition, implies unstained cells; the second quadrant represented annexin V-FITC only positive cells undergo early apoptosis; the third quadrant represented both annexin V-FITC and PI positive cells undergo late apoptotic; whereas the fourth quadrant represented PI-positive only cells undergo necrosis.

**Animal model.** Male C57BL/6 mice (also known as C57BL/6J, 6- to 8-week-old) and Male athymic nude mice (BALB/c nu/nu, 6- to 8-week-old) were supplied by the Laboratory Animal Center of Zhejiang Chinese Medical University. All mice were housed in colony cages at 25 °C with 12 h light/12 h dark cycles, with 45% relative humidity. All experiments involving animals (License No. IACUC-20200611-05) were performed with the approval of and in accordance with the guidelines of the Institutional Animal Ethics Committee of Laboratory Animal Center of Zhejiang Chinese Medical University.

**In vivo antitumor efficacy in MC38 tumor models.** To establish tumor models, cells were harvested using 0.25% Trypsin-EDTA solution, washed with PBS, counted and resuspended in PBS. For the allograft experiment, murine colorectal carcinoma MC38 cells or bioluminescent MC38 cells (MC38-Luci) that stably express luciferase (1 × 10^6^, 100 µL) were subcutaneously injected into the right flanks of C57BL/6 mice. Mice were randomized to six groups when tumors reached a mean volume of approximately 70~90 mm^3^: CPT11 (SN38 equivalent of 5 mg/kg), CPBSN38L (SN38 equivalent of 5 mg/kg, DSPE-PEG-Ce6 equivalent of 25 mg/kg), CL+US (DSPE-PEG-Ce6 equivalent of 25 mg/kg), PBSN38L+US (SN38 equivalent of 5 mg/kg), CPBSN38L+US (SN38 equivalent of 5 mg/kg, DSPE-PEG-Ce6 equivalent of 25 mg/kg) and the control group (PBS). The treatment was delivered by intravenous injection every 3 days for a total of 5 times. US stimulation (intensity: 0.8 W/cm^2^, 3MHz, 50% of duty cycle, duration: 5 min, Mettler Sonicator-740) will used at 6 h after intravenous injection of drugs. Body weight, tumor size and animal condition were monitored at regular intervals. Tumor volume was calculated using the formula: Tumor Volume (mm^3^) = (smallest diameter^2^ × largest diameter) / 2. The tumor growth curves are displayed as mean ± SD versus days after the first treatment. Differences between groups were compared with ordinary one-way ANOVA. Mice were sacrificed 21 days after the first treatment. Resected tumors and major organs were weighted and fixed in 4% paraformaldehyde fix solution for 24 h before embedding in paraffin. The inhibition rate of tumor growth (IRT) was calculated as follows: IRT = 100% × (mean tumor weight of the control group – mean tumor weight of the experimental group) / mean tumor weight of the control group.

**Biodistribution and in vivo, ex vivo imaging****.** MC38-Luci tumor-bearing mice were used to evaluate the biodistribution and tumor accumulation of CPBSN38L in vivo. When tumors reached about 400 mm^3^, the mice were intravenously injected with CPBSN38L^Cy5^ via the tail vein. The fluorescent signal was observed at an excitation of 640 nm and emission of 710 nm at 12 h after injection. The mice were sacrificed at termination, tumors as well as major organs including heart, liver, spleen, lung, kidneys, intestine were collected, weighed, and subjected for ex vivo imaging using the same parameters described above. The real-time distribution of liposomes and quantitative analysis of the fluorescent intensity were recorded by the IVIS Lumina II (PerkinElmer, USA).

**Histological examination and immunofluorescent analysis of tumor tissues.** The excised tumors and major organs were fixed in 4% paraformaldehyde fix solution, paraffin embedded and sectioned into 5 µm slices. The sections were stained with hematoxylin-eosin (H&E) and imaged using an inverted bright field microscope for histological examinations. For Ki67 staining, paraffin sections were deparaffinized, antigen retrieval, washed, permeabilized and blocked with 5% BSA and further incubated with anti-Ki67 antibody (Abcam, 1:250) at 4 °C overnight. Corresponding Alexa Fluor® 488 anti-rabbit secondary antibody (CST, 1:1000) and DAPI were then used for staining for confocal imaging to assess cell proliferation in each group. TUNEL was performed using Invitrogen BrdU TUNEL Apoptosis Assay kit according to the manufacturer’s protocol.

**In vivo antitumor efficacy in Huh7 tumor models and** **assessment of** **treatment-associated toxicity.** To establish tumor models, cells were harvested using 0.25% Trypsin-EDTA solution, washed with PBS, counted and resuspended in PBS. For the xenograft assay, human liver cancer Huh7 cells (5 × 10^6^, 100 µL) were subcutaneously injected into the right flanks of nude mice. Mice were randomized to six groups when tumors reached a mean volume of approximately 70~90 mm^3^: CPT11, topotecan (TPT), CL, CPBSN38L, CPBSN38L (SN38 equivalent of 5 mg/kg, DSPE-PEG-Ce6 equivalent of 25 mg/kg) and the control group (PBS). US stimulation (intensity: 0.8 W/cm^2^, 3MHz, 50% of duty cycle, duration: 5 min, Mettler Sonicator-740) will used at 6 h after intravenous injection of drugs in all groups. The treatment was delivered by intravenous injection every 3 days for a total of 5 times. Body weight, tumor size and animal condition were monitored at regular intervals. Tumor volume was calculated using the formula: Tumor Volume (mm^3^) = (smallest diameter^2^ × largest diameter) / 2. The tumor growth curves are displayed as mean ± SD versus days after the first treatment. Differences between groups were compared with ordinary one-way ANOVA. On day 21, whole blood was anticoagulated with EDTA for the blood routine in mice to evaluate the chemotherapy-induced myelosuppression using automated hematology analyzer (Sysmex, USA). At the same time a portion of the blood was collected without EDTA in EP tubes. After 30 min resting, the supernatant serum was separated via 3000 rpm centrifuged for 10 min at 4 °C for biochemistry testing using full-automatic biochemical analyzer (HITACHI, Japan). Then mice were sacrificed for histopathological examinations. Full-length intestines were excised and fixed in 4% paraformaldehyde fix solution. The entire intestine was rolled and embedded in paraffin and intestinal cross-sections through intestinal rolls spanning the entire intestines were prepared for further histopathological and apoptosis evaluation. The sections were stained with H&E and imaged using an inverted bright field microscope for histological examinations. TUNEL was performed using Invitrogen BrdU TUNEL Apoptosis Assay kit according to the manufacturer’s protocol.

**Statistical analysis**

Each experiment was performed at least three times in triplicates. Data were presented as mean ± SD (standard deviation of the mean). Grouping was by random selection and each picture were selected from random microscopic fields. Graphing and all statistical analyses were performed with GraphPad Prism 9 and OriginPro 2020. Statistically significant differences were assessed by one-way analysis of variance (ANOVA) with Tukey’s correction and 95% confidence intervals for categorical variables. *p* value <0.05 considered to be statistically significant.

**Supporting Schemes and Figures**





**Scheme S1**. Synthesis of the prodrugs: **A** phenylboronic acid pinacol ester-conjugated SN38 (PBSN38), and **B** phenylcarbinol-conjugated SN38 (PSN38), and their ROS-responsive activation.


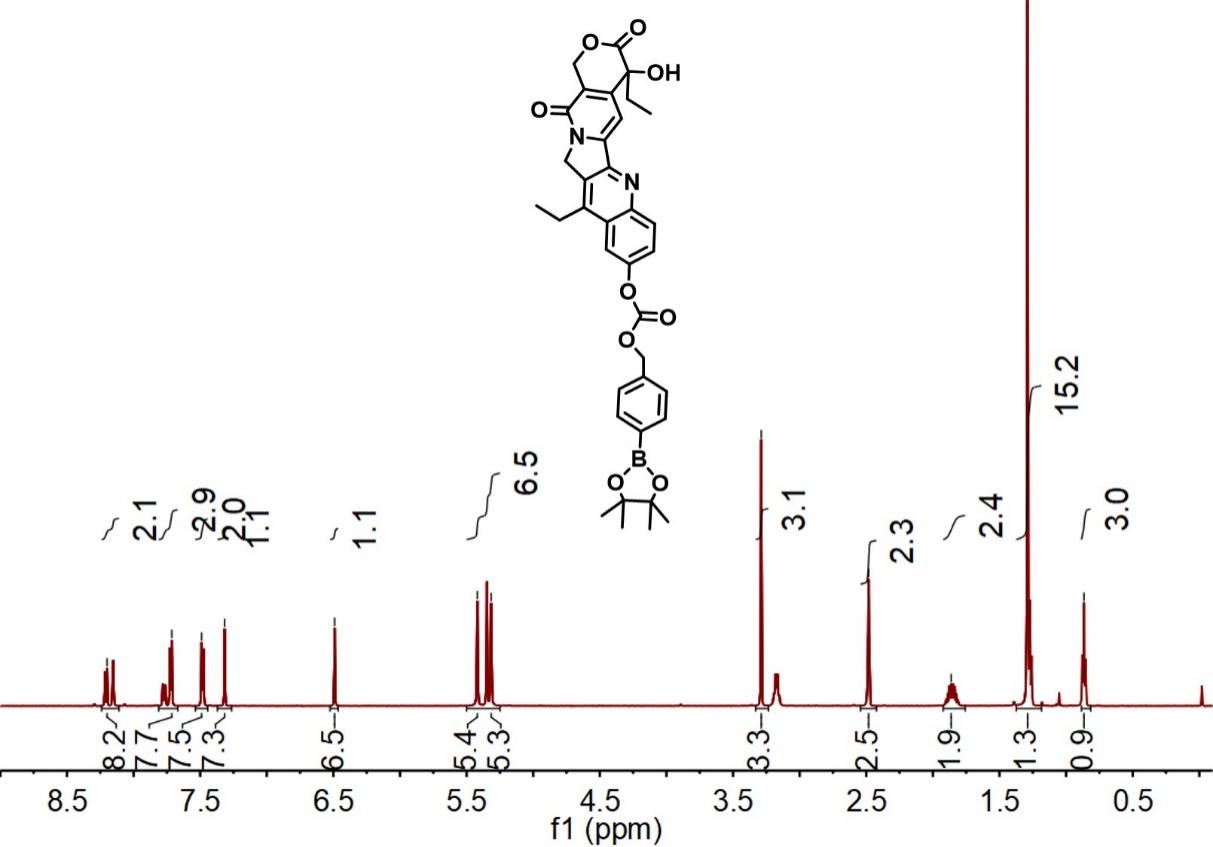


**Figure S1.** The ^1^H-NMR spectrum of PBSN38 in CDCl_3_.


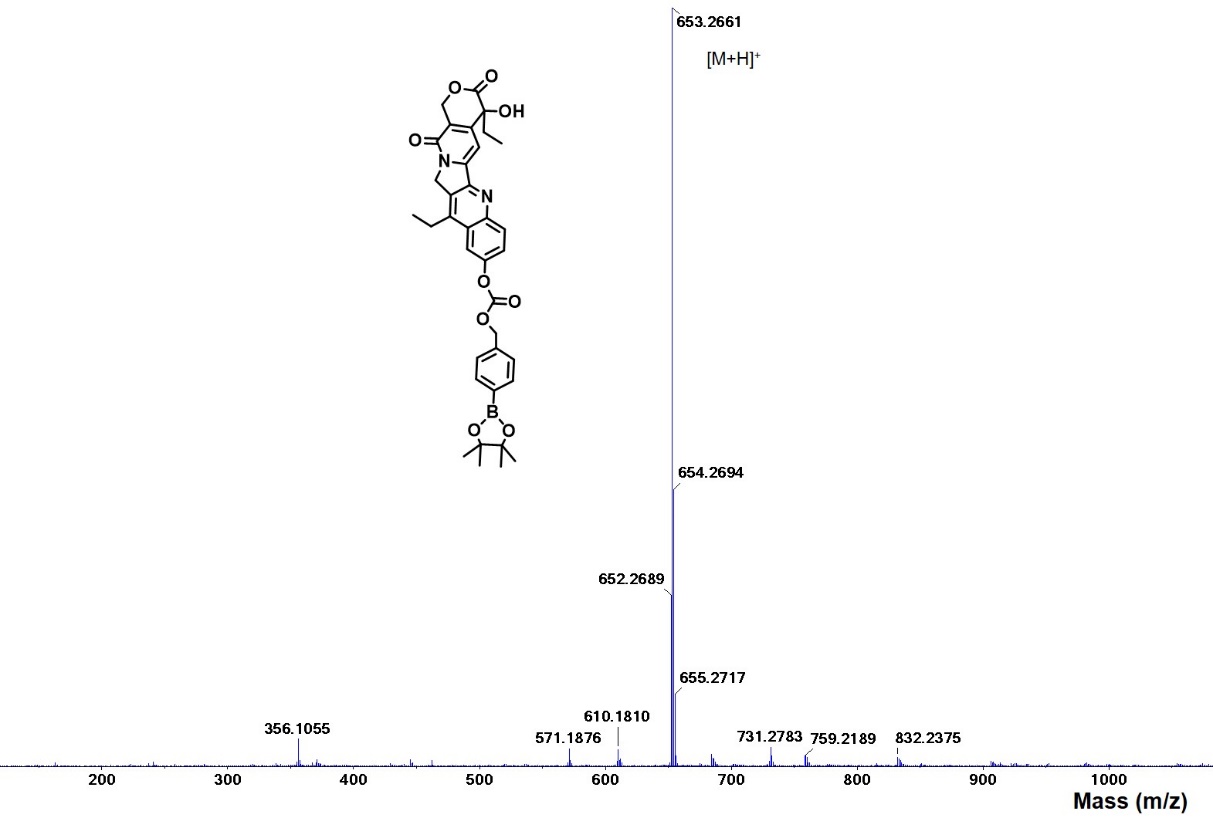


**Figure S2.** The MALDI-TOF-MS spectrum of PBSN38.


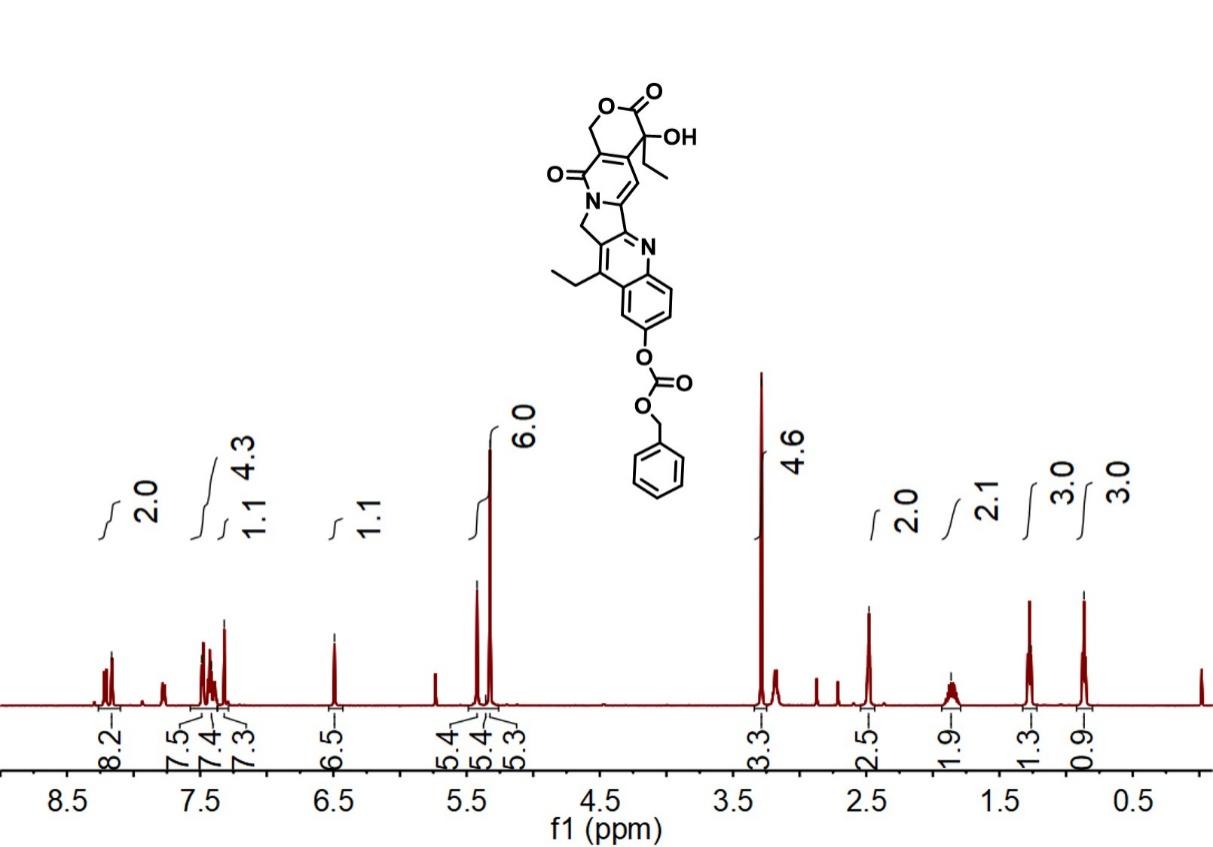


**Figure S3.** The ^1^H-NMR spectrum of PSN38 in CDCl_3_.


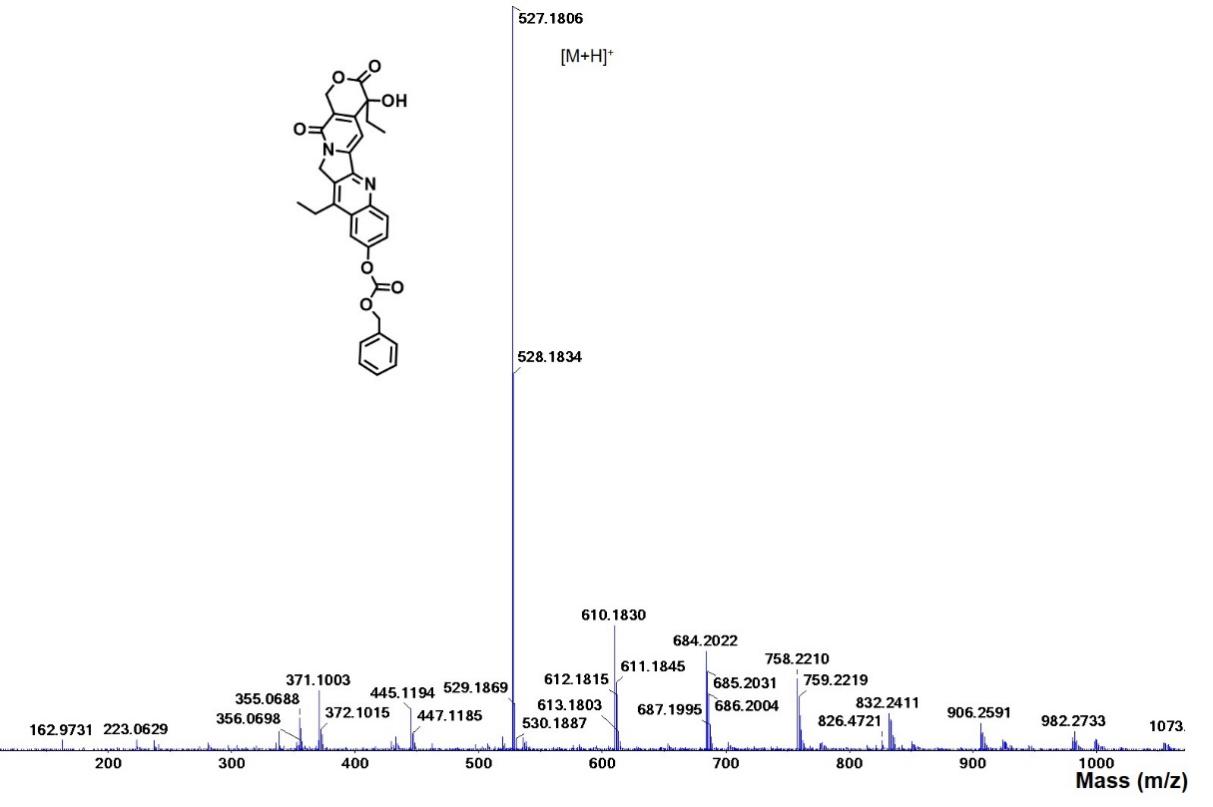


**Figure S4.** The MALDI-TOF-MS spectrum of PSN38.


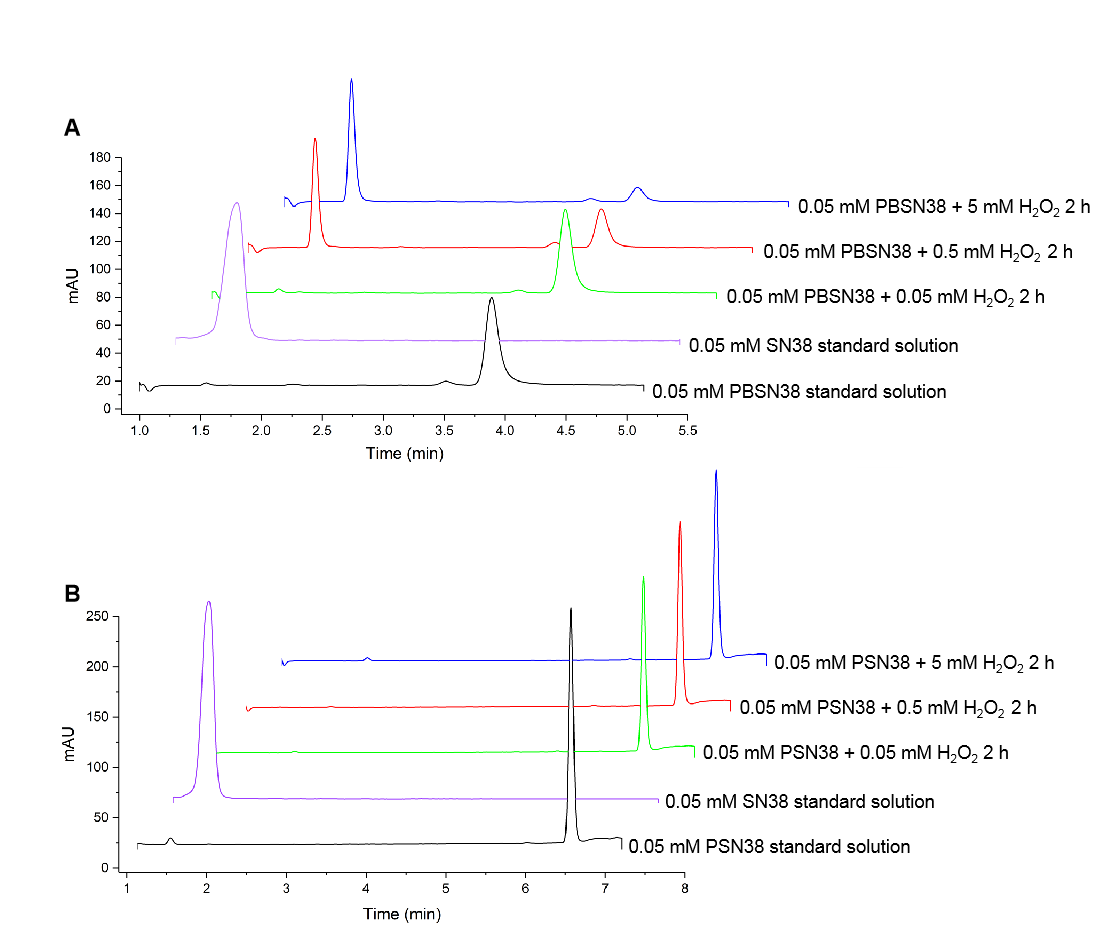


**Figure S5.** **Drug activation of PBSN38 and PSN38 were monitored by tracking the SN38 releasing.** **A** PBSN38 (0.05 mM) was incubated with different concentrations of H_2_O_2_ in methanol solution at 37^o^C for 2 h. The samples of PBSN38 were analyzed using the Agilent ZORBAX Eclipse XDB-C18 column (75 × 4.6 mm, 3.5 µm) at 40^o^C in Agilent-1100. The mobile phase was eluted at 1.0 mL/min and consisted of acetonitrile/H_2_O (35:65, v/v). The detector was set at 254 nm. **B** PSN38 (0.05 mM) was incubated with different concentrations of H_2_O_2_ in acetonitrile and methanol mixture solution at 37^o^C for 2 h. The samples of PSN38 were analyzed using the mobile phase of methanol/H_2_O (30:70, v/v).


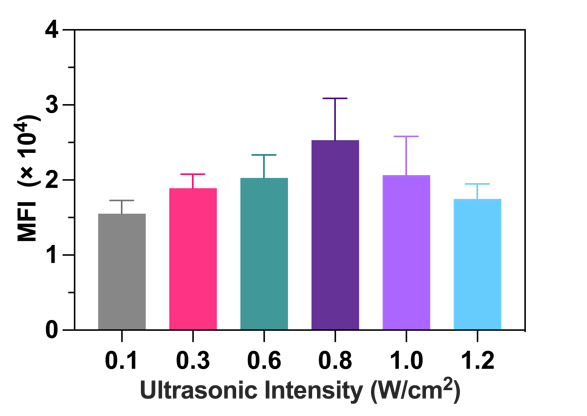


**Figure S6.** Quantitative results of the flow cytometry analysis of the intracellular ROS levels in MC38 colon adenocarcinoma cells treated with CPBSN38L under different acoustic intensity.


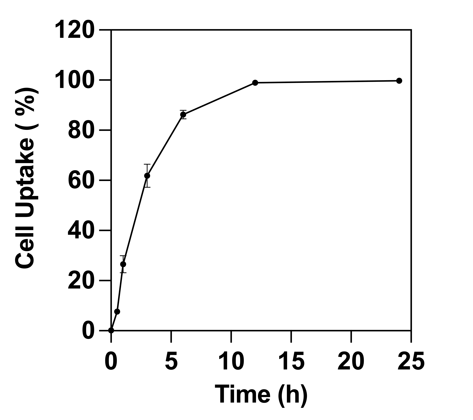


**Figure S7.** The flow cytometry analysis of cellular uptake rate of CPBSN38L in different time points.


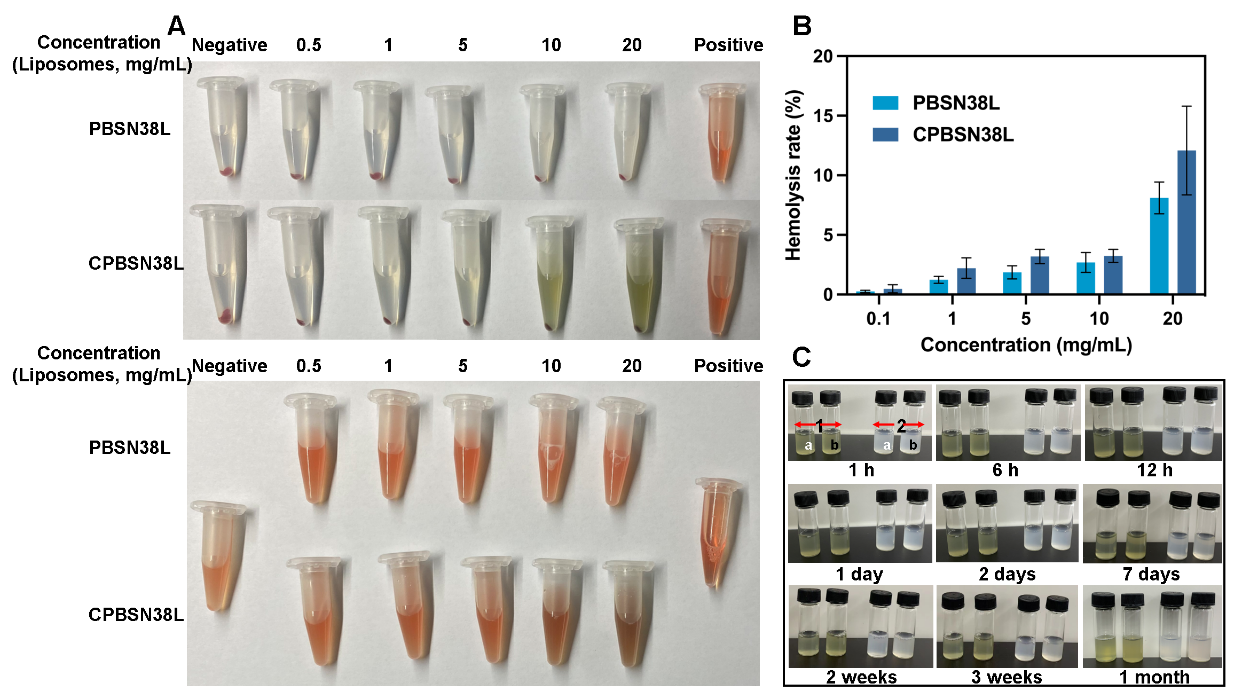


**Figure S8.** **In vitro hemolysis was performed using mouse red blood cells after incubation with the liposomes.** **A** Photographs of RBC suspensions after 2 h incubation in a shaker at 37 °C with PBSN38L and CPBSN38L at the lipid concentration in the range of 0.1-20 mg/mL. **B** Hemolysis rate of the liposomes at different concentrations. The saline was set as negative (0% hemolysis) and the 1% Triton X-100 was set as positive (100% hemolysis). **C** Photographs of *(1)* CPBSN38L and *(2)* PBSN38L in PBS containing (a) 0% or (b) 10% FBS at room temperature for one month.


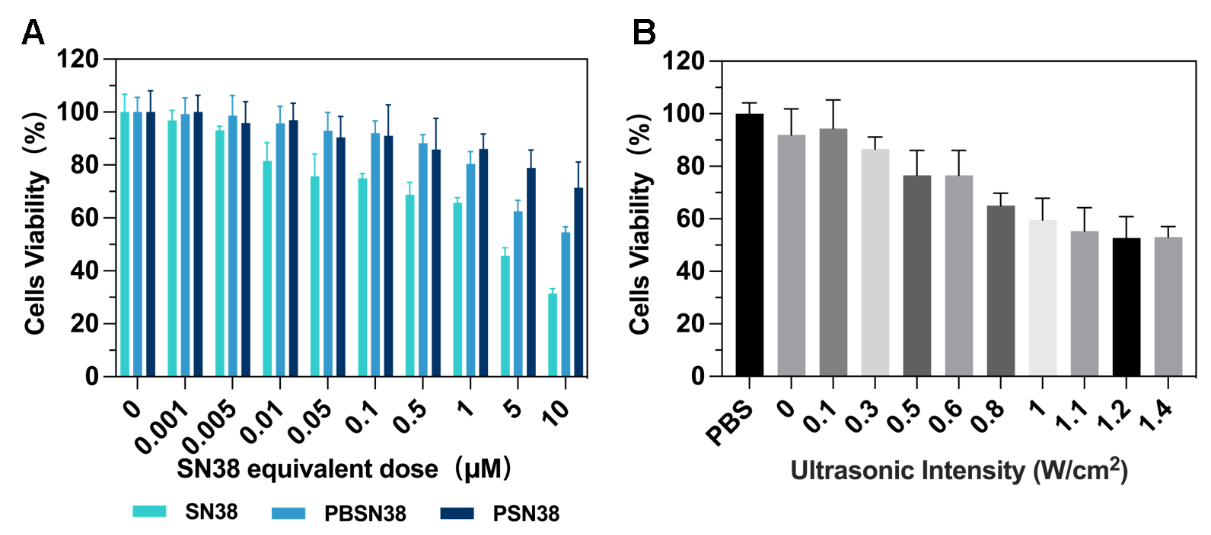


**Figure S9.** **In vitro cell viability.** **A** The cell viability of MC38 was detected by CCK-8 kit after incubation with free drug of SN38, or the prodrug of PBSN38 and PSN38 without US irradiation. **B** The cell viability of MC38 was tested after incubation with 15 µM Ce6 and treatment with US irradiation (US parameter: 3 MHz, 50% of duty cycle for 5 min).


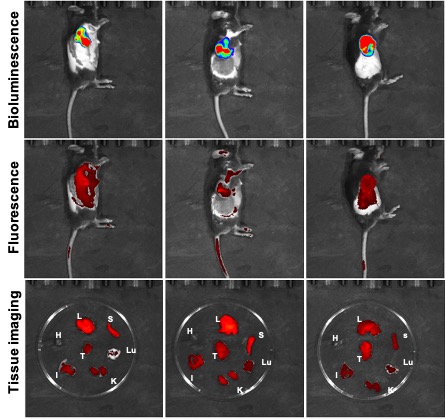


**Figure S10.** **In vivo biodistribution of CPBSN38L.** Representative bioluminescence images and fluorescence images in mice whole body or the excised organs (T: tumor, H: heart, L: liver, S: spleen, Lu: lung, K: kidney, I: intestines).


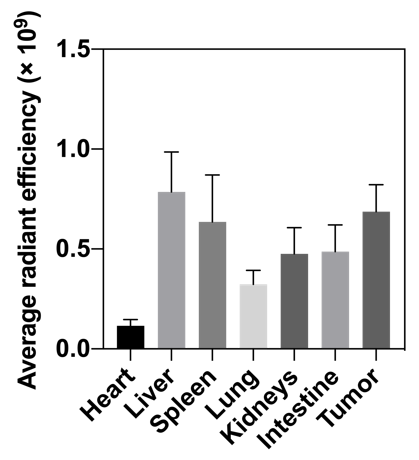


**Figure S11.** The fluorescence intensity of CPBSN38L in different organs and tissues were quantified with the unit of radiant efficiency using the Living Image^®^-4.5 software.


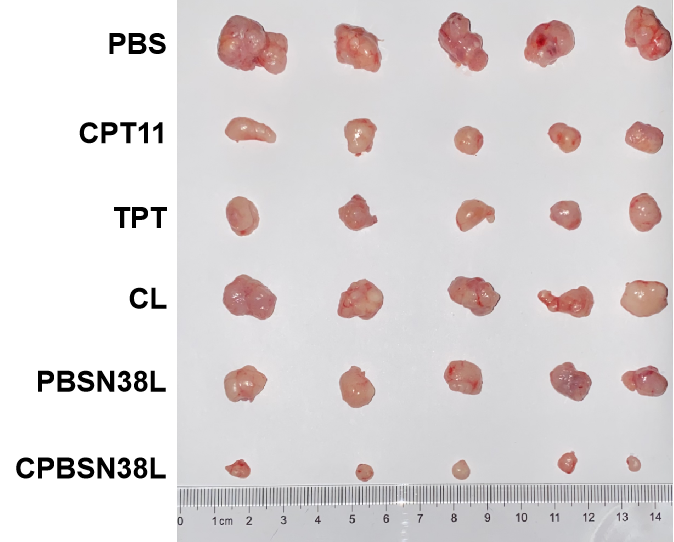


**Figure S12.** The photograph of resected Huh7 tumors in different groups.


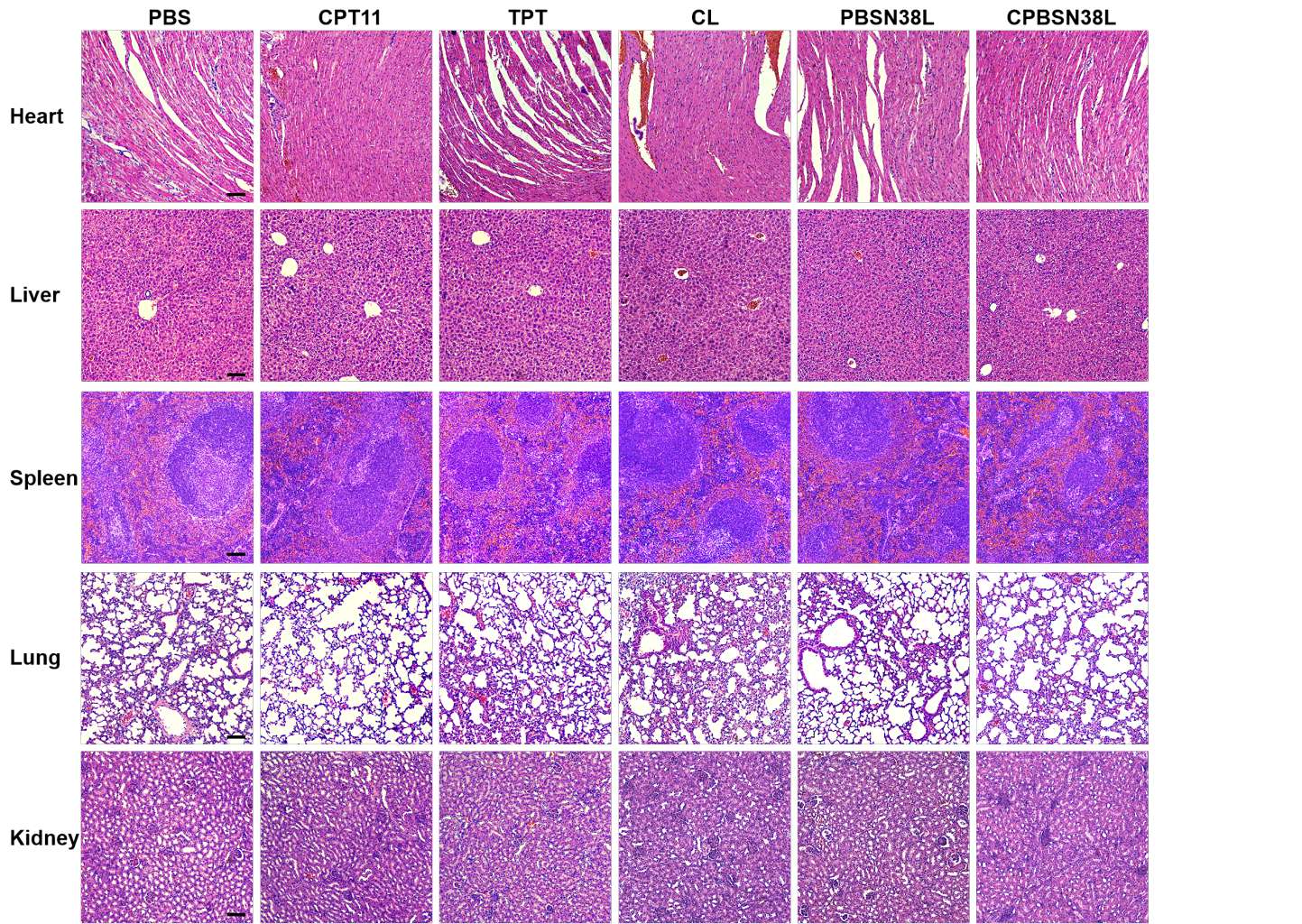


**Figure S13.** Representative images of the H&E staining of main organ sections in each group. All groups were treated with US irradiation (0.8 W/cm^2^, 3 MHz, 50% of duty cycle, 5 min) at 6 h after intravenous injection of drugs. Scale bar = 100 µm.

**REFERENCES**

1. Drummond DC, Noble CO, Guo ZX, Hong K, Park JW, Kirpotin DB. Development of a highly active nanoliposomal irinotecan using a novel intraliposomal stabilization strategy. Cancer Res. 2006;66:3271-7.

2. Wibroe PP, Ahmadvand D, Oghabian MA, Yaghmur A, Moghimi SM. An integrated assessment of morphology, size, and complement activation of the PEGylated liposomal doxorubicin products Doxil (R), Caelyx (R), DOXOrubicin, and SinaDoxosome. J of Controlled Release. 2016;221:1-8.
